# A checklist of European butterfly larval foodplants

**DOI:** 10.1002/ece3.10834

**Published:** 2024-01-07

**Authors:** Harry E. Clarke

**Affiliations:** ^1^ Surrey UK

**Keywords:** Europe, habitat, host plants, Lepidoptera, Papilionoidea

## Abstract

Butterflies are charismatic insects and have been well studied, particularly in Europe. They are disproportionately used in generating and testing hypotheses; on everything from general evolutionary processes, such as speciation or host association dynamics; to conservation‐related studies, such as climate change or habitat loss. Accurate lists of the larval foodplants for European butterflies are not readily available. Mistakes are propagated and information cannot be checked for accuracy. The level of evidence is unknown, and how usage varies between countries is poorly understood. The study consulted 1119 references to produce 19,488 records of larval foodplants for European butterflies. This resulted in 5589 larval host plant records for 464 European butterfly species, with multiple references, enabling information to be checked. Information was unavailable for 59 species. The level of evidence for each relationship shows the current state of knowledge. Significant issues were identified for 3.9% of records extracted from references due to mistakes, ambiguous or unknown plant names, distribution issues, resulting in information being lost. Plants with questionable distributions suggest either mis‐identification or species that have been split. Little is known about plant usage in Eastern Europe. The larval foodplants of many monophagous and Satyrinae butterflies are poorly studied. Only 63% of threatened 2010 Red Listed butterflies have reliable host plant records. The study has provided ecologists with a valuable resource, of a more accurate checklist of the larval foodplants for each European country. Why plant usage varies over a butterfly's distribution opens up some interesting research questions.

## Graphical abstract

The study consulted 1119 references to produce 19,488 records of larval foodplants for European butterflies. This resulted in 5589 larval host plant records for 464 European butterfly species, with multiple references, enabling information to be checked. Significant issues were identified for 3.9% of records extracted from references due to mistakes, ambiguous or unknown plant names, distribution issues, resulting in information being lost.

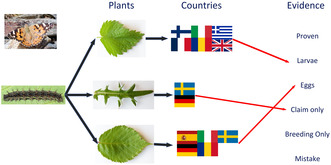

## 1 INTRODUCTION

While butterflies are not the most economically important, they are charismatic insects and have been well studied particularly in Europe, with an unusually large amount of information available. For this reason, butterflies are disproportionately used in generating and testing hypotheses—on everything from general evolutionary processes, such as speciation or host association dynamics to conservation‐related studies, such as the effects of changes in climate or land use. Consequently, it is crucial that the data used in such studies are correct.

The larval stage of butterflies is the stage where the main resources are consumed. The resources are typically the leaves, stems, flowers or seeds of angiosperms, although members of the *Phengaris* genus in later instars feed on ant brood. Each plant species has particular habitat requirements which can be defined by a number of different attributes, such as those defined by Ellenberg et al. (1991) and Grime (2001). Ecological indicator values for European plants have been produced (Dengler et al., 2023; Tichý et al., 2023).

Evolutionary ecologists need detailed information on larval foodplant usage to understand how the relationship between plants and butterflies has evolved (Ehrlich & Raven, 1964). It is known that plants produce anti‐herbivore measures such as secondary chemicals (Schoonhoven et al., 2005). Larvae need to overcome these defences to enable them to attain maturity. Different plant species deploy different anti‐herbivore measures. Consequently there is a strong link between butterfly families and the plant families that they utilise. The usage of different plant species in different regions or habitats could indicate cryptic speciation. For example, Hinojosa et al. (2023) suggested that the use of different plants has led to reproductive isolation for *Eumedonia eumedon* (Esper, 1780). Alternatively, plant availability or plant quality may be significant. Information on the distribution of plants (POWO, 2023) and butterflies (Wiemers et al., 2018) is readily available. However, accurate information on plant usage is not readily available.

Species are under threat from climate change and habitat loss, and hence are in need of conservation efforts to prevent further loss. Conservationists need accurate information on the resource requirements, and what the limiting factors are, to enable them to know whether conservation efforts will be successful or not. Why butterflies use different plants in different regions and habitats has been poorly studied. For example, climatic conditions may alter a plant's suitability, as suggested by Nylin et al. (2008) for *Polygonia c‐album* (Linnaeus, 1758).

Many lists of larval host plants have been produced in the past (e.g. Tolman & Lewington, 2008; Tshikolovets, 2011). However, these lists contain many errors, Blab and Kudrna (1982) warn of the mistakes that have been made in the past and the original work cannot always be checked for technical accuracy, so false information continues to be used, which can lead to wrong conclusions. Brower (1958) undertook a critical review of the foodplant preferences for five butterflies in the *Papilio glaucus* group and identified a number of issues with the claimed larval foodplants.

A previous review of the European larval foodplants (Clarke, 2022) identified a number of unanswered questions. What evidence is there for the host plant usage beyond a claim in a single reference? Does host plant usage vary throughout the butterfly's range or is usage constant? Are all host plants for a particular species used equally or does usage vary?

As with all scientific information, the original source needs to be checked for technical accuracy. The question that needs to be answered is can the larva complete its development on this particular plant species, and is this particular plant used in the wild. However, there are complications. Members of the *Phengaris* genus feed on ant brood in later instars. Two species are known to be able to swap plant species. *Euphydryas maturna* (Linnaeus, 1758) post‐diapause larvae can utilise different plants to pre‐diapause larvae (Dolek et al., 2012), and *Pyrgus centaureae* (Rambur, 1839) can switch from *Betula nana* L. to *Rubus chamaemorus* L. (Wickman, 2012). Hence, the question needs to be revised to can the larva complete its normal part of its development on that plant.

The aim of the study is to summarise the current state of knowledge of the larval foodplants of European butterflies. The study was limited to Europe due to resource and data constraints; Western Europe has been particularly well studied, so multiple primary references should increase confidence in the accuracy of the data. The study will attempt to answer the following questions: (1) Is the host plant usage constant throughout Europe, or does it vary from country to country? (2) What level of evidence is available for that usage? (3) Is the claimed plant usage consistent with the known distributions of the butterfly and plant? (4) Is the claimed plant usage consistent with the butterfly's known biology?

## 2 MATERIALS AND METHODS

### 2.1 Search methodology

Two strategies were deployed to obtain information on larval foodplants. First books providing lists of larval foodplant usage for each country. And, second, publications providing primary evidence (oviposition, eggs or larvae found in the wild) for larval foodplant usage. Breeding records were included to support usage in the wild records, or where there was limited information on a particular butterfly species. ABBYY FineReader and Google Translate were used to help with the translation of non‐English sources.

Books on the butterflies of a country often provide a list of the larval foodplants, which the authors believe are used in that country. Often they make use of unpublished information and also can provide a useful list of references to be checked. Searches were made for both English and the native language book titles. The AbeBooks and WorldCat websites were searched for countries that appeared to be without a relevant book.

Google Scholar was used to search for primary information on the larval foodplant usage, for each butterfly species. The scientific name was the main search criteria, along with other search criteria such as larvae, eggs and oviposition. A search was also made of some print‐only journals, such as Entomologist Gazette, Linneana Belgica and Alexanor. Often when a particular source was checked, while not providing primary evidence itself, provided a list of references to be checked for larval foodplants. All publications providing primary evidence found during searches were included.

References were confined to permanent publications (e.g. books and journals) so that the information can be checked. This excludes ephemeral information, such as that which is only available online (e.g. Lepiforum, 2023). References were restricted to those that specified a country or a small region in Europe (e.g. Fennoscandia). A few exceptions were allowed (Feltwell, 1982; Tutt, 1906, 1908, 1909, 1914), which have not materially affected the results.

In a few cases, it was not possible to obtain copies of all potential references. This applied particularly to books that were unavailable new, or on the second‐hand market. And to print‐only journals of local societies that had not been digitised and were not held by the Royal Entomological Society library.

### 2.2 Geography

There is no formal definition of Europe, with different authors providing a slightly different definition (Brummitt, 2001; Collins Bartholomew Ltd, 2018; Wiemers et al., 2018), as it is part geographical and part political. Initially it was decided to use the countries as defined in Wiemers et al. (2018). However, to allow checking of the distribution of plants, the regions defined in Brummitt (2001) at Level 3 were used. The key difference between the two lists is that European Russia is divided up into smaller regions, and the Mediterranean islands of Corsica, Sardinia, Sicily and Crete are listed separately from the countries they belong to. For this study, European Russia includes all of Russia west of the Urals, which includes the Caucasus.

### 2.3 Database

Given the size of some of the tables and to implement constraints on the data in the tables to minimise errors on data entry, a PostgreSQL database was created, consisting of seven tables (deposited in Dryad Data Repository), and a further three tables of plant information imported from external sources (see below). The main table ‘larval_plants’ contains the records of the information extracted from each publication. The original information in the publication was retained. The published scientific name (the vernacular name only included to aid in plant identification), the assumed scientific name (see below for details) and the currently accepted name. Thus for each butterfly and plant there are three names, linked by the various tables. Each record records the life stage that was observed in the wild (oviposition, eggs and larvae) and the usage of the plant where known (primary, secondary, rarely and breeding). A degree of judgement was required, as the language used by publications did not always make it clear whether the information was a claim or was based on personal observation.

The table ‘larval_host_plant’ contains the accepted butterfly and plant names, along with the countries, references and evidence for each plant used by the butterfly. The evidence uses all the information for the particular plant/butterfly combination on a six‐point scale (Table 1). It is arguable whether breeding should have a higher or lower score than a claim. It is known that some claims are based on unpublished work of usage in the wild, but they do not provide the details. Breeding experiments are used for many different purposes and often use locally available plants, especially for the grass feeders, even though these plants are not known to occur in the habitat occupied by the larvae in the wild. Breeding experiments combined with evidence of usage in the wild have been scored as proven. The evidence for each plant‐butterfly relationship is thus the lowest overall score from all the records. Although multiple references for a particular plant obviously carry more weight than a single reference and could be considered as equivalent to proven, even though breeding experiments have not been carried out, they have been scored as eggs or larvae as appropriate in this study.

**TABLE 1:** Definition of the evidence for plant usage.

No.	Evidence	Description
1	Proven	Plant used in the wild and proven by breeding
2	Larvae	Larvae found in the wild on the plant
3	Eggs	Oviposition or eggs found in the wild on the plant
4	Claim only	Claimed utilisation in the wild, but without evidence
5	Breeding only	Plant used in breeding experiment
6	Mistake	Larvae do not survive on plant or insufficient evidence

### 2.4 Butterflies

There is no known publicly available comprehensive database of butterfly synonyms. A table ‘butterfly_synonym’ was created to include all butterfly names encountered in the publications and link those names to the currently accepted name. Lamas (2008) was used to provide the generic names of butterflies and their synonyms. Since the publication of Wiemers et al. (2018), further research has revealed new species. For the records of larval foodplants, it is better to provide finer detail when species are split, as these may be significant. For example, in the split of *Muschampia proto* (Ochsenheimer, 1808) (Hinojosa et al., 2021), records could be allocated to the new species of *M. alta* (Schwingenschuss, 1942), *M. proto* and *M. proteides* (Wagner, 1929), as their distributions are allopatric. However, in the splits of *Leptidea sinapis* (Linnaeus, 1758; Dincă et al., 2011; Lorkovic, 1993), this was not possible, as the distribution of *L. sinapis*, *L. reali* Reissinger, 1990 and *L. juvernica* Williams, 1946 is sympatric; older records of *L. sinapis* have to be allocated to *Leptidea sinapis* s.l., as identification of the specific species is unknown, except in a few cases, such as in England and Ireland. As a constraint on this checklist of larval foodplants, records not identified to species or subspecies are excluded from the analysis, although they are included in the raw data.

The full checklist of the 523 European butterfly species used in this study, along with the changes to the checklist of Wiemers et al. (2018), is provided in the Appendix S3. Various resources were used to identify the full scientific name of the synonyms encountered in the sources consulted, in particularly Bozano (2023), funet (2023) and GBIF (2023). In a few cases, the date of publication of the taxon name could not be identified.

Species that have been split or renamed were dealt with by creating subspecies of the species name specified in the resource. For example, the European species now known as *Melitaea ornata* Christoph, 1893 was originally called *M. telona* Fruhstorfer, 1908 (Tóth et al., 2012). A synonym was created of *M. telona ornata*, with the accepted species *M. ornata*. Thus European records of *M. telona* are linked to the assumed butterfly name of *M. telona ornata*.

No taxonomic decision was made on subspecies names, even if some subspecies seemed suspect, for example, *Parnassius apollo* (Linnaeus, 1758). All defined subspecies of an accepted species were treated as accepted, unless they were a synonym for another species. In the analysis of the records, the butterfly subspecies records were merged with the species records.

### 2.5 Plants

The International Plant Names Index (Govaerts et al., 2022; IPNI, 2023) provides the list of all plant names that have been formally published but does not provide any taxonomic decisions. Plants of the World Online (POWO, 2023), which is working in collaboration with World Flora Online (Borsch et al., 2020; Miller et al., 2022; WFO, 2023), provides current accepted plant names. Version 11 of the World Checklist of Vascular Plants (WCVP), names and distribution (Govaerts et al., 2021), was downloaded from the POWO website. All the plant names in the IPNI have a unique ID (ipni_id), which is used in other databases, such as POWO and WFO.

Plant names used in the publications consulted caused many issues, such as misspelling, failure to provide taxon authors or only providing a vernacular name. These issues if not identified and resolved can lead to incorrect conclusions been made. The issues encountered are explained below.

Misspelling of the taxon name was resolved where possible, by using Google to search for the correct name. Misspelling of the taxon authors were resolved manually, by checking the various options for the taxon name.

Ambiguity of plant names occurs when the same taxon name has been used by several different authors to refer to different species, and the taxon authors are omitted from the publication. For example, in WCVP there are nine records with the taxon name *Rubus tomentosus*, all with different authors. Of these nine records, three are for different species and six are currently unplaced. Fortunately, only *Rubus tomentosus* Willd. has a European distribution enabling the plant to be uniquely identified. However, for *Potentilla grandiflora* there are seven records with different authors; of which four records are unplaced and three records are for different species, all with a European distribution. *P. grandiflora* L. is the currently accepted name, *P. grandiflora* M.Bieb. is an illegitimate name for *P. crantzii*, (Crantz) Beck ex Fritsch and *P. grandiflora* Scop. is an illegitimate name for *P. tommasiniana* F.W.Schultz. In such cases, it is unclear which species is being referred to, and it cannot be assumed that the currently accepted name is the correct one. For example, when taxon authors are specified, Lafranchis et al. (2015) used *Sedum montanum* Perrier & Song. and Deschamps‐Cottin et al. (1997) used *Sedum montanum* L. as larval foodplants for *Parnassius apollo* L. in France. The currently accepted names for those two plant species are *Petrosedum montanum* (Songeon & E.P.Perrier) Grulich and *Sempervivum montanum* L., respectively.

Misuse of plant names occurs when a published name is being used for one species, but the name was published for a different species. For example the plant name *Ulmus campestris* L. has been used by various authors (e.g. Barrett, 1893; Frohawk, 1924a, 1924b; Tutt, 1908). These authors were using *Ulmus montana* to refer to Wych Elm and *Ulmus campestris* to refer to Common or English Elm. While *Ulmus montana* Stokes was correctly been used to refer to Wych Elm, as it is a synonym for *U. glabra* Huds.; however, *U. campestris* was being misused for Common Elm, as it is also a synonym for *U. glabra*. The accepted scientific name for Common or English Elm is *U. minor* subsp. *minor*. Without the inclusion of the vernacular name, it would be very easy to assume the wrong species, or to not know the plant name was being misused. Confusingly, Ebert and Rennwald (1991a, 1991b) uses *Ulmus glabra* ‐ Berg‐Ulme, *Ulmus campestris* and *Ulmus minor*. Whereas Gozmány (1968) and Lorković (2009) just use *Ulmus campestris*, without providing further details.

Abuse of plant names (illegal name) occurs when a name is being used for a plant species that has not been published (i.e. without description nor type specimen). For example, the name *Iberis linifolia* Loefl. has been used in scientific papers (e.g. Huertas Dionisio, 1986, 1989). The reference quoted in the Euro+Med PlantBase (2023) is ‘Iter Hispan.: 78. 1758’ (Loefling, 1758), but that does not provide a description of the species, just a list of plant names. The Euro+Med PlantBase states that the name *Iberis linifolia* Loefl. is a synonym for *Iberis contracta* Pers. subsp. *contracta*, which POWO states has an Iberian distribution, and this is consistent with the usage in the publications consulted However, GBIF states (source: The Leipzig catalogue of vascular plants) *Iberis linifolia* Loefl. is a synonym for *Lepidium linifolium* (Desv.) Steud., which POWO states has an Australian distribution. It is possible that Loefling (1758) was referring to *Iberis linifolia* L., which Linnaeus published in 1759. However, without a description or type specimen, it is pure speculation which species was being referred to.

To resolve these issues, a table was created (wcvp_extendedb) which provided a mapping between the plant name recorded in the larval plant record, and the WCVP table of plant names. For example, when there was misuse of a plant name, such as for *Ulmus campestris* L., it can be mapped to *U. minor* subsp. *minor*. For each name in the table, the name status was recorded with the following definitions:

- Published is a plant name published in IPNI and in the WCVP database.
- IPNI is a plant name published in IPNI but not yet included in the WCVP database. GBIF and WFO provided synonyms for these names in the WCVP database, except for two species.
- WCVP is a plant name in the WCVP database, which has not been formally published.
- Ambiguous is a plant taxon name that has multiple authors referring to different species within Europe. All plants recorded as agg. are treated as ambiguous. If the ambiguous names are within the same genus, a link can be provided to the WCVP database. The larval foodplant records with ambiguous names, which are in different genera, cannot be used in this work.
- Illegal is a plant name that has not been formally published, and not in the WCVP database, but can be assumed to be a synonym for a published name in the WCVP database. The notes column provides the details.
- Misuse is a plant name used for a different species to that of the type specimen and description.
- Unknown is a plant name which is an unknown synonym of any known species. The larval foodplant records are consequently lost.

The following rules were used for inclusion of plants as larval foodplant records. Plants must be identified to genus, species, subspecies, variety or form to be included. If the plant scientific name included the taxon author, then that must agree in part or full with the plant author in the checklist, allowing for variations in spelling. If the taxon author was excluded (unfortunately a common feature of many publications), the following rules were applied

- If the taxonomic name was unique, it was assumed to be the species, subspecies, variety or form being referred.
- If the taxonomic name had multiple authors, but the known plant distribution enabled the plant species to be uniquely identified. For example, *Prunus spinosa* L. has a European distribution, whereas *P. spinosa* Walter has an American distribution.
- If the taxonomic name had multiple authors, which were all synonyms of the same plant, the accepted author was used. For example, *Filipendula vulgaris* Hill is a synonym of *F. vulgaris* Moench.
- If the taxonomic name had multiple authors, and the publication cited a flora used, the flora was checked to determine the plant author. The following floras were consulted:
  ○ Sonderegger (2005) used the flora Lauber and Wagner (1996a, 1996b).
  ○ LSPN (1987) and (1999) used the flora Binz and Heitz (1986).
  ○ Ebert and Rennwald (1991a, 1991b) used the flora Oberdorfer (1983).
- If the taxonomic name had multiple authors and a vernacular name was provided, then an internet search was used to determine the full scientific name usually associated with that vernacular name.
- If the taxonomic name had multiple authors, and the particular species, subspecies, variety or form could not be uniquely identified, the name was defined as Ambiguous.
- Generic names were assumed to be the accepted genus name, even if the name was ambiguous.

### 2.6 Checks

Checks were made to ensure that the butterfly name and the plant name were linked to the correct assumed name. The distribution of butterflies was checked against that of Wiemers et al. (2018), and subsequent species splits, and where possible distribution issues resolved to the correct species.

The distribution of plants was checked against the WCVP database. The table of larval host plants contains two columns; one with records of countries with a reasonable distribution, and a second with records of countries with a questionable distribution. Records with a questionable distribution issues are likely to be identification errors, or where a species has been split.

Checks were made that the larval foodplants were reasonable for the butterfly's known biology. Plant species that were in a different plant order or family without sufficient evidence were marked as mistakes. APG IV (2016) was used to assign plant families to orders. Generally these were egg or claims only records where the plant order was not used by other members of the same butterfly tribe. Some judgement was required, as there are a few butterfly species which are truly polyphagous on many different plant orders. As when additional evidence comes to light, those records marked as mistake can be re‐evaluated.

## 3 RESULTS

A total of 19,488 records of the larval foodplants of European butterflies from 1119 references (Appendix S2) were obtained. 149 records could not be used because the plant name was either unknown or was ambiguous with species in different genera. Table 2 provides a breakdown of the 2444 different plant names that were used in the references. Ambiguous species are included in the genus or unknown plant name rank. There are four plant species, whose accepted scientific name in WCVP does not have a link to the IPNI, representing work in progress (*Rubus communis* Bayer; *Lophiolepis ferox* (L.) Del Guacchio, Bureš, Iamonico & P.Caputo; *Lophiolepis eriophora* (L.) Del Guacchio, Bureš, Iamonico & P.Caputo; *Oxytropis neglecta* subsp. *neglecta*).

**TABLE 2:** Plant names used in references.

Plant name rank	Number
Genus	361
Species	1888
Subspecies	123
Variety	19
Unknown	53
Total	2444

**FIGURE 1:**
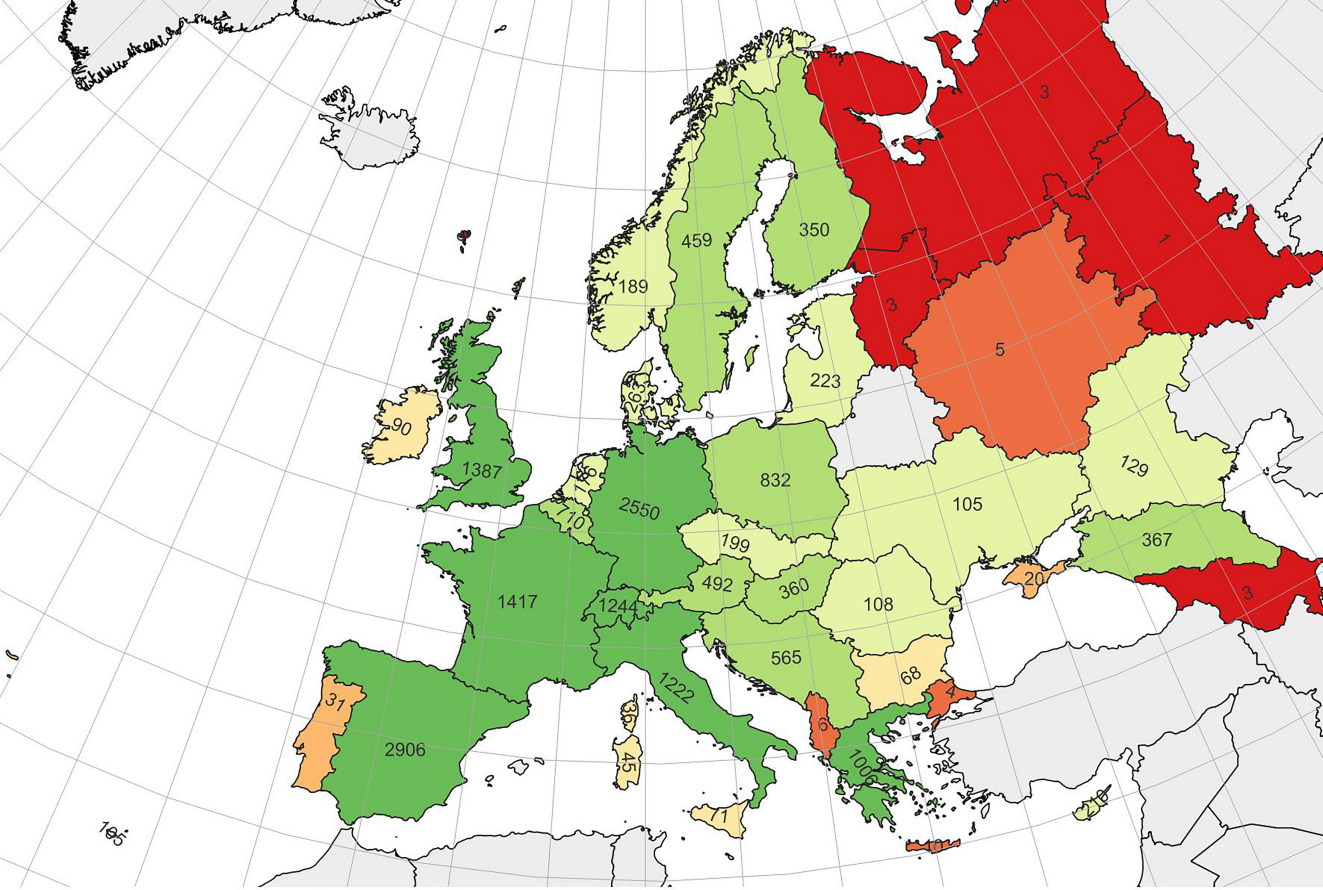
Heatmap of the number of larval foodplant records for each region in Europe.

The number of records per country varied enormously (Figure 1), with Germany, Spain, France, United Kingdom, Switzerland, Italy and Greece, each with over 1000 records. However, there are 38 countries or regions with <100 records. Northern and Western Europe have better coverage than Eastern Europe.

The abuse and misuse of plant names specified in books and scientific literature is shown in Table 3, with nearly 8% of plant names unknown in either IPNI or WCVP. An astonishing 4.5% of plant names cited in the references were ambiguous in WCVP, and a further 1.1% unknown, which means that important information is being lost. The misuse of plant names, unless detected, is a case of assigning the wrong plant species as a larval foodplant.

**TABLE 3:** Taxon status of plant names used in references.

Plant taxon name status	Number	Percentage
Published	2200	90.0
IPNI	20	0.8
WCVP	34	1.4
Ambiguous	109	4.5
Illegal	49	2.0
Misuse	6	02
Unknown	26	1.1
Total	2444	100

These records resulted in 5589 larval foodplant relationships for 464 European butterfly species and 1972 different plants (Appendix S1). Information on the larval foodplants for 59 butterfly species could not be obtained. A checklist of the 523 butterfly species used in this study is provided in Appendix S3. The records of de Tré (1987) was found to be unreliable, as many records are suspect and are not supported by other authors.

**FIGURE 2:**
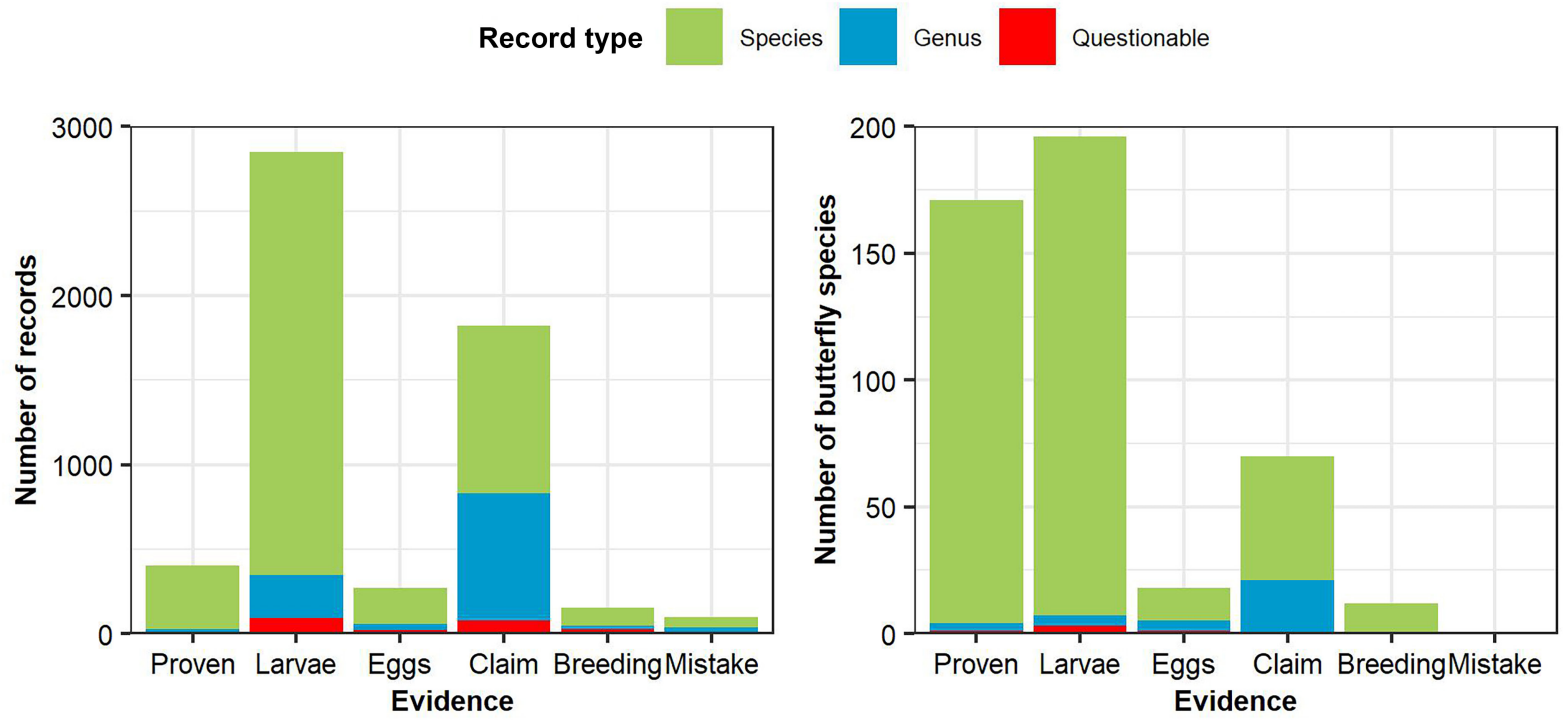
Number of records and butterfly species with different levels of evidence with one or more plant species or genera.

In interpreting the results, the evidence given is based on the references for that particular taxonomic name and not at lower taxonomic levels. For example, this may mean a generic name has evidence of ‘Claim only’, while there are species in that genus which may have evidence of ‘Larvae’ or ‘Proven’. Figure 2 provides a breakdown of the evidence provided for each record. These results show that 92% of records have a claim of usage in the wild, excluding plants with a questionable distribution (197 records), mistakes (95 records) and breeding only (154 records). And only 55% of larval foodplant records can be considered reliable, assuming all the records of usage in the wild are correct and correctly identified to species level or lower. Four per cent of larval host plants have a questionable distribution, which suggests plant identification issues or taxonomic splits, and therefore must be considered unreliable.

Figure 2 also shows the number of European butterfly species with their highest level of evidence for larval foodplant. This shows that only 69% of butterfly species have at least one robust larval foodplant record; larval or proven records with a reasonable plant distribution.

Mis‐identifications and taxonomic splits are issues in any taxonomic group. The grass *Festuca ovina* L. is the most widely recorded plant species, used by 71 butterfly species (this study). However, this is probably a mis‐identification of *Festuca ovina* agg. as *Festuca ovina* L. is not found in Albania, Azores, Iberian Peninsula, Sardinia and Scilly, as recorded for 16 butterfly species. *Festuca ovina* agg. is a group of about 20 closely related species, which requires specialist knowledge to identify correctly.

The number of different plant species, genera, families and orders used by each European butterfly species, with reliable records, is provided in Appendix S4. Usage varies greatly from one plant species to 78 (*Vanessa cardui*) and from one plant order to 14 (*Celastrina argiolus*). When butterfly species are grouped by family, subfamily, tribe and subtribe, clear patterns of plant families and order usage are shown (Appendix S5). These patterns of plant specialisation on particular plant families is to be expected (Downey, 1962; Gilbert & Singer, 1975). However, some results can confuse the picture, due to some very polyphagous butterfly species which can utilise plants in many different plant orders outside the plant orders normally used other butterflies in the same tribe.

This study has shown that larval foodplant usage does vary from country to country, although the reasons are beyond the scope of this study. For example, *Aglais io* (Linnaeus, 1758) is only known to use *Urtica urens* L. in Spain and the United Kingdom, and *Parietaria judaica* L. in Greece, while *Humulus lupulus* L. and *Urtica dioica* L. are widely used in many European countries.

The claimed plant usage is consistent with the known distributions of the butterfly and plant. Records identified with a questionable butterfly distribution (e.g. due to species splits) have been linked to the correct species. Records with a questionable plant distribution have been identified in a separate list.

The claimed plant usage is more consistent with the butterfly's known biology. By identifying the plant order and family, claims of usage (without larval evidence) in a different plant order have been identified as mistakes. For example, *Hamearis lucina* (Linnaeus, 1758) larva feed on various plants in the family Primulaceae, order Ericales. Oviposition on plants in different plant orders is unlikely to be larval foodplants and have been marked as mistakes.

**FIGURE 3:**
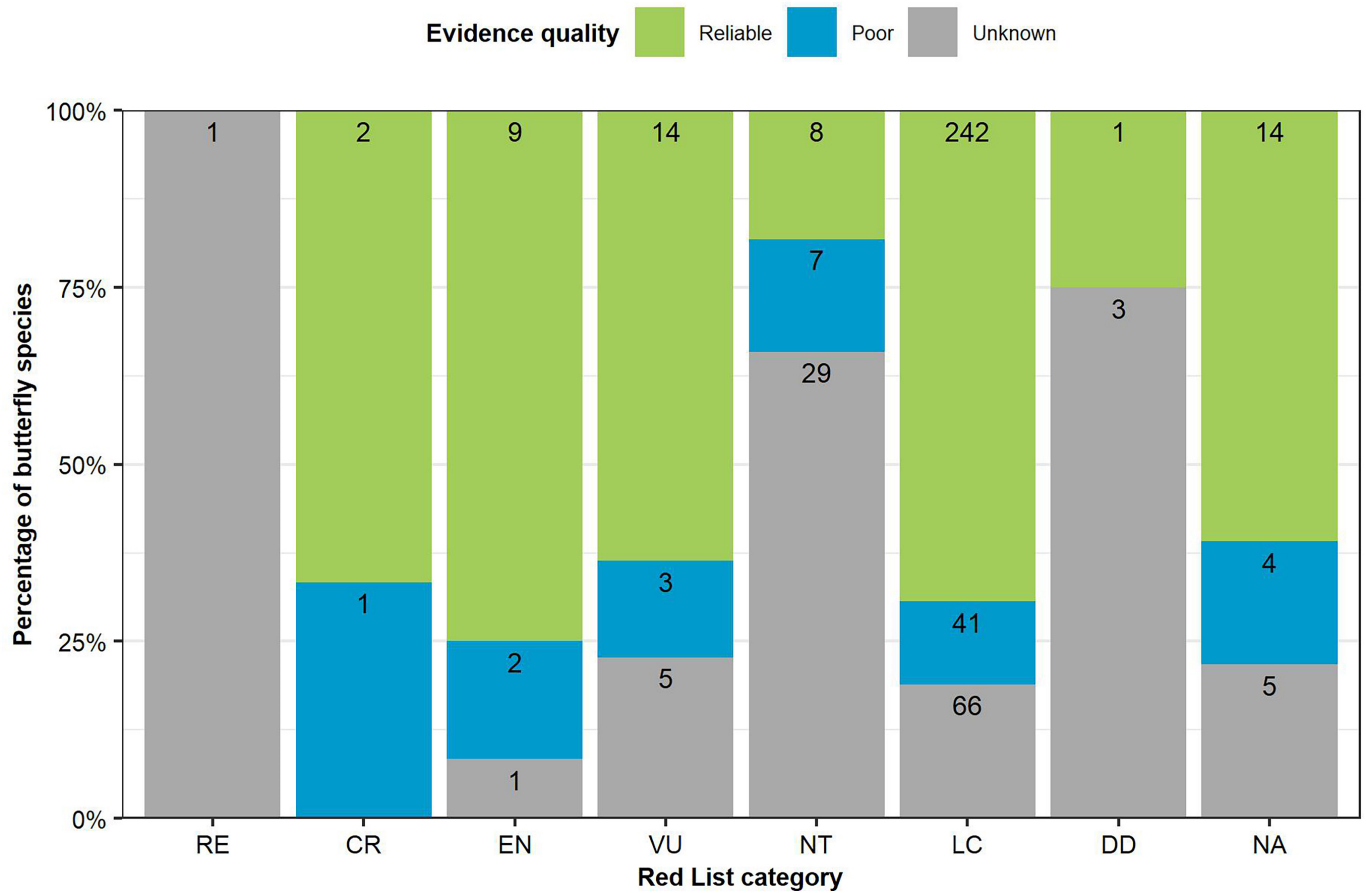
Evidence for 2010 Red List butterfly larval foodplant usage, providing the number of butterfly species in each category.

An analysis of what is known of the larval foodplants of 481 Red Listed butterflies (van Swaay et al., 2010), is shown in Figure 3. Appendix S6 provides the evidence for each butterfly species in the Red List, along with its current accepted name. *Polyommatus galloi* (Balletto & Toso, 1979) is now considered a subspecies of *Polyommatus ripartii*, and *Polyommatus pljushtchi* (Lukhtanov & Budashkin, 1993) is now considered a subspecies of *Polyommatus damone*. Only 63% of threatened butterfly species (CR, EN and VU) have reliable evidence (plant usage in the wild with a reasonable distribution) for larval host plants, and 21% of threatened butterfly species have unknown evidence (breeding only or unknown).

## 4 DISCUSSION

A more accurate checklist of the larval foodplants for 464 butterfly species has been provided for each European country, with mistakes and questionable distributions identified. Information was unavailable for 59 species. Primary references are provided so that information can be checked. The level of evidence for each relationship between host plant and butterfly species provides the state of current knowledge. The butterfly and plant distributions have been checked to be reasonable. The plant usage is now more consistent with the known biology of the butterfly, with obvious mistakes identified. This will provide a valuable resource for future researchers, saving much time in searching for relevant literature and highlighting where there are gaps in our knowledge.

The main issues encountered were the poor use of plant names and the lack of knowledge in many countries. The use of unknown, ambiguous plant names or mistakes resulted in information being lost (1.4% records extracted from references). The citing of plants outside their known distribution (2.5% records extracted from references) is probably due to mis‐identification of the species or later species splits, resulting in unreliable records which will require further research to clarify. The lack of knowledge in many countries not only highlights where further research is required but also means the wrong conclusions may be made.

### 4.1 Breeding

Breeding experiments are used for a multitude of different reasons, not all of them are useful for confirming larval foodplant usage. Breeding experiments are required to demonstrate that larvae can complete their normal development on that plant species, and that observations in the wild are from genuine larval foodplants, and not being used for some other purpose (e.g. pupation, movement between plants, etc.). However, breeding experiments rarely replicate the environment that larvae encounter in the wild. This can have an impact on the results. Temperature effects larval digestion and growth (Heinrich, 1993, 1996). Daylength and temperature effects when larvae enter and exit diapause (Danilevskii, 1965; Gill et al., 2017). But these factors also affect how plants respond in their production of secondary chemicals (Cipollini, 2002; Schoonhoven et al., 2005). To produce meaningful breeding results, the conditions the larvae experience in the wild need to be replicated in the lab as closely as possible.

Positive breeding results demonstrate that the larva is able to metabolise the plant chemicals so that it can grow and complete its development on that plant. However, negative results are more difficult to interpret. This may be a plant on which the larva has a poor survival rate on that plant, and further breeding experiments could confirm this. Or that the larva only use plants with specific secondary chemicals which differ from plants of the same species growing in the area (Singer et al., 2002).

### 4.2 Monophagous, oligophagous and polyphagous feeders

There is no formal definition of the terms monophagous, oligophagous and polyphagous, with different authors using slightly different definitions. The term oligophagous was introduced by Brues (1920) with the definition of ‘several definitely fixed ones’ The results show for butterflies with reliable records (usage in the wild and a reasonable distribution) that there is a spectrum from utilising just one plant species (61 butterfly species), to one plant genus (146 butterfly species), one plant family (276 butterfly species), one plant order (314 butterfly species) and more than one plant order (64 butterfly species). There are nine butterfly species where the plant names are only known at the generic level.

**FIGURE 4:**
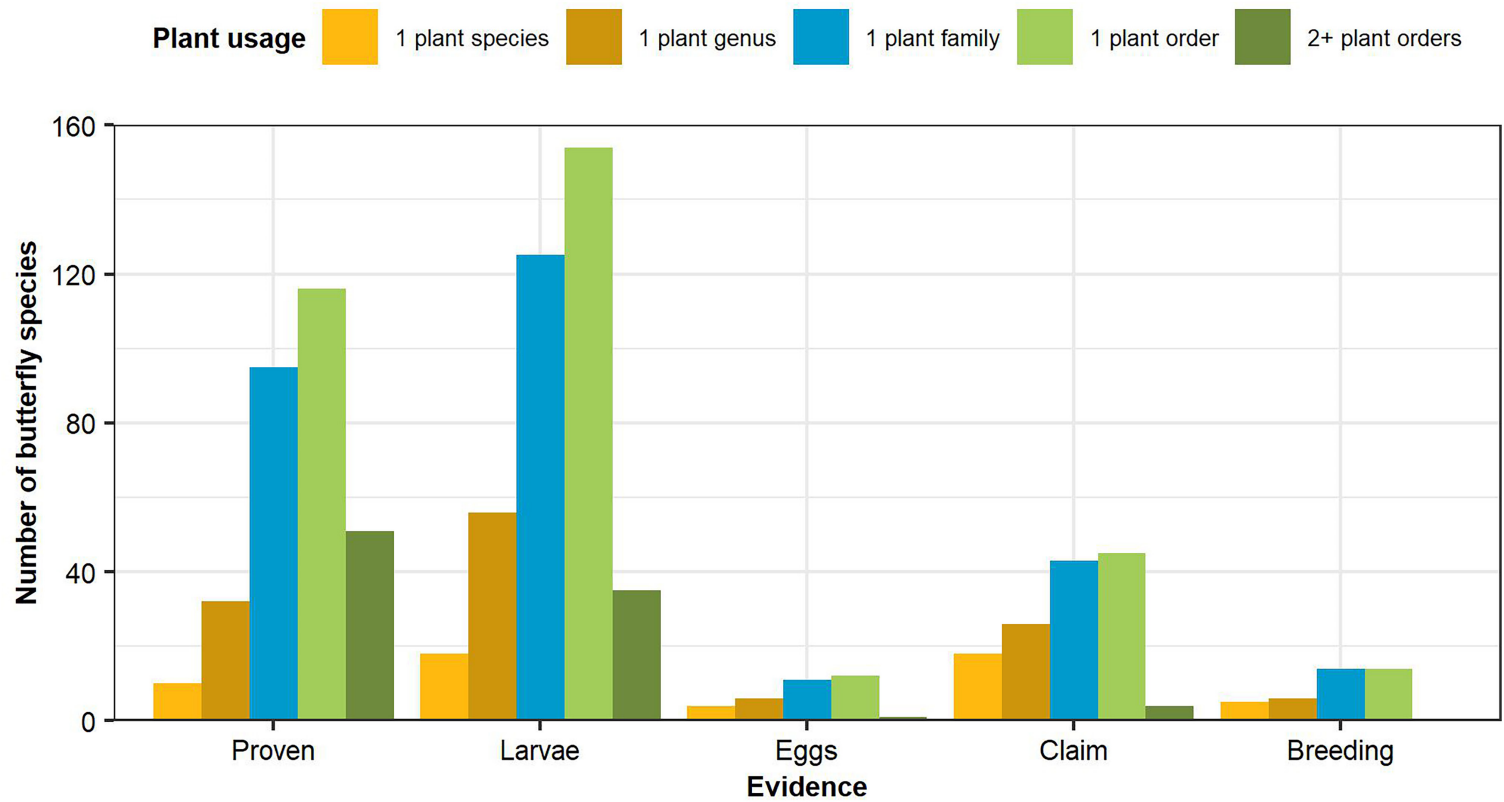
Level of evidence for different number of plants utilised by European butterfly larvae.

It would seem reasonable to define monophagous as utilising larval host plants in one plant genus (39% of European butterfly species), oligophagous as utilising larval host plants in more than one genus in one plant family (34% of European butterfly species) and polyphagous as utilising larval host plants in more than one plant family (27% of European butterfly species). 17% of the latter ones are polyphagous on more than one plant order. This is a slightly different definition to that used by Cates (1981) who defined oligophagous to additionally include ‘or closely related families’. Figure 4 shows the evidence available for the 432 European butterfly species with at least one known plant species with a reasonable distribution.

**FIGURE 5:**
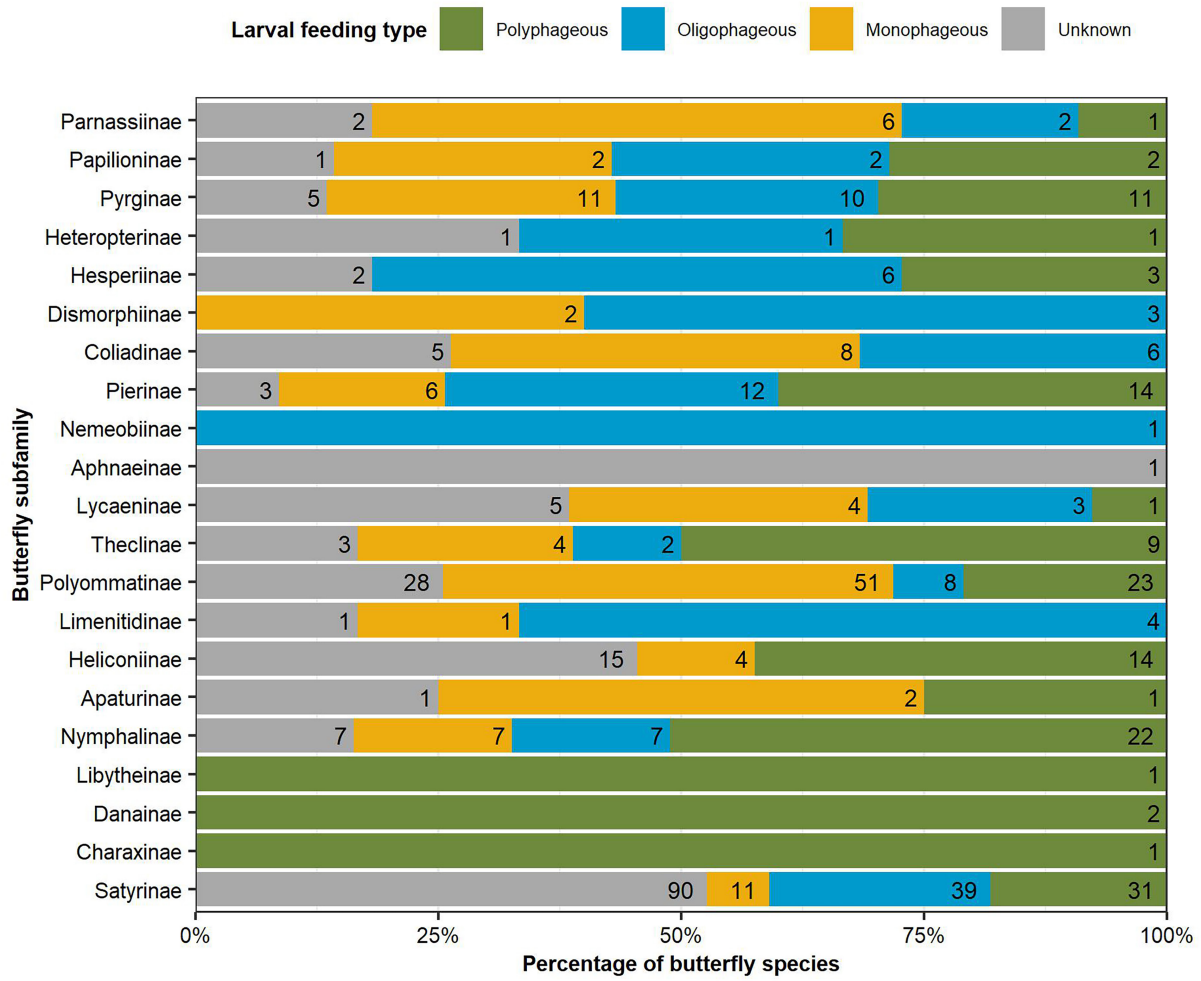
Knowledge of butterfly subfamily larval host plants from monophagous to polyphagous, providing the number of butterfly species in each category. Reliable records only.

The larvae of European butterfly species utilise 80 different plant families, which updates the number given by Munguira et al. (2009). The most important ones are Poaceae, Fabaceae, Rosaceae, Cyperaceae, Brassicaceae, Asteraceae, Lamiaceae, Violaceae, Polygonaceae and Plantaginaceae. However, 20 plant families are only used by one butterfly species. Figure 5 shows what is known about the larval host plants of the butterfly subfamilies. 362 species can be classified as monophagous, oligophagous or polyphagous with reliable information, and 170 butterfly species as unknown. Only 56% of known monophagous butterfly species have reliable larval host plant records. Whereas there is far better information available for polyphagous species, with 95% with reliable larval host plant records.

### 4.3 The grass feeders

Poales are used by three butterfly subfamilies: Heteropterinae, Hesperiinae and Satyrinae. Primarily they use the plant family Poaceae and less often the families Cyperaceae and Juncaceae. The primary anti‐herbivore defences used in grasses are silica concentration, phenolic concentration and leaf toughness (Massey et al., 2007). Grasses that use C_4_ photosynthesis are harder to digest (Schoonhoven et al., 2005). The impact on butterfly larvae is that the silica wears down their jaws (Massey & Hartley, 2009) and reduces their growth rate and digestion efficiency (Massey et al., 2006).

Grass‐feeding larvae can be difficult to study, as they are often cryptically coloured, hidden during the day and only emerge in the cover of darkness to feed (Joy, 1996). For example, for years it was thought that the larval foodplant of *Pseudochazara williamsi* (Romei, 1927) was a *Festuca* (Munguira et al., 1997). However, it is now thought this is a mis‐identification of the grass *Koeleria vallesiana* (Honck.) Gaudin. Despite decades of study, oviposition has never been observed, nor larvae found, but this grass is always found in the butterflies' habitat, and the grass seems more suitable for the larvae (Javier Oliver, personal communication).

Very little research has been undertaken on the larval foodplants of these Poales feeders, even though they represent 35% of European butterfly species. Many questions remain unanswered. For example, do these larvae feed on just one species of grass during their development, or do they swap between different species? What are the limiting factors that determine which species of grass are used? The nutritional value of the plant, such as nitrogen and carbohydrate concentrations? Or the silica content? How do the environmental factors impact on the anti‐herbivore response of the plant?

Soil pH and temperature seems to be important for some species. For example, in England, *Hesperia comma* (Linnaeus, 1758) is monophagous on *Festuca ovina* L. on calcareous grasslands (Bennie et al., 2013). However, this is probably a mis‐identification of *F. ovina* subsp. *ophioliticola* (Kerguélen) M.J.Wilk., which prefers calcareous soils, whereas *F. ovina* prefers acidic soils (Stace, 2019). *F. ovina* subsp. *ophioliticola* is a synonym for *F. ophioliticola* Kerguélen.

### 4.4 Plant usage

Plants and butterflies have evolved together. Plant chemistry is known to be an important factor regarding the suitability of plants for herbivores. Anti‐herbivore measures deployed by plants are expensive to produce and are generated at the expense of plant growth (Schoonhoven et al., 2005). Plants limit the production of secondary chemicals when they are most effective: These secondary chemicals can vary between individual plants (Singer et al., 2002), they depend on the age of the plant, the location of the plant (e.g. sunny or shady), time of day, the soil the plant is growing in or even determine whether adjacent plants are being attacked by herbivores (Schoonhoven et al., 2005).

**FIGURE 6:**
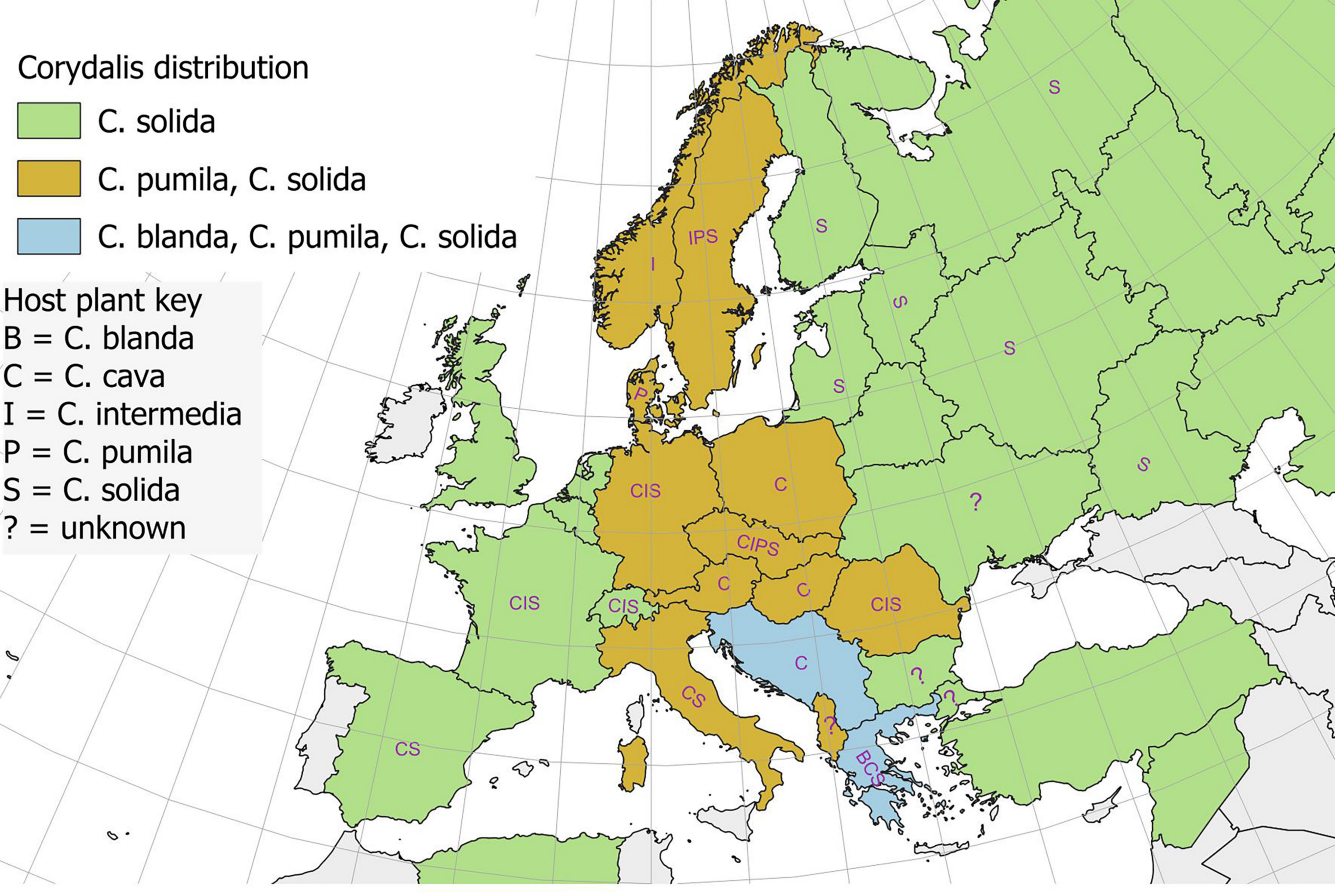
*Parnassius mnemosyne* larval foodplants in Europe.

While the larval foodplant use of butterflies has been studied (e.g. Appendix S5), the question why butterfly larvae utilise different plants in different regions or habitats has been poorly studied. For example, *Parnassius mnemosyne* (Linnaeus, 1758) has records for seven larval foodplants in Europe (Appendix S1), but two of these are claims only. Figure 6 shows which host plants are used throughout its range where larvae have been found in the wild. Three of these plants are widespread throughout Europe, with *Corydalis solida* (L.) Clairv. appearing to be the primary larval host plant. The other two plants, *C. pumila* (Host) Rchb. and *C. blanda* Schott, have limited distribution in Europe (Figure 6). The host plant usage raises a number of interesting questions, which can only be briefly be explored here.

Could the larval foodplant usage of *P. mnemosyne* be explained by its evolutionary history? Gratton et al. (2008) investigated its evolutionary history for the central European populations and showed there were several different lineages. This may explain the usage of *C. blanda* in Greece. More detailed research would be required before any conclusions could be drawn, taking into account the western European populations, and matching those with larval foodplant usage.

Plant quality may explain why *C. pumila* is only used in northern countries, with plants in the south having stronger anti‐herbivore defences which the larvae cannot overcome. Lack of research in the countries of the former Yugoslavia may explain the lack of records for *C. blanda*, but this does not explain the lack of usage of *C. pumila* in Italy. Another possibility is that the plants may be particularly scarce in the habitats occupied by the butterflies in those countries.

## 5 CONCLUSION

This study has shown that even for a taxonomic group that has been well studied, especially in Europe, and the impression given by earlier lists, only 69% of European butterflies have good evidence for one or more larval foodplants. Major gaps in our knowledge have been identified. Monophagous and Satyrinae butterflies have been poorly studied. Only 64% of threatened 2010 Red Listed butterflies have reliable larval foodplant records. Plant usage in Eastern Europe is poorly known. This study provides ecologists with a valuable resource of a more accurate list of butterfly larval foodplants for each European country and the ability to check that information. Why plant usage can vary over a butterfly's distribution remains an interesting research question.

## AUTHOR CONTRIBUTION

**Harry E. Clarke:** Writing – original draft (equal).

## FUNDING INFORMATION

The author has funded this study.

## CONFLICT OF INTEREST STATEMENT

The author declares no conflict of interest.

## Supporting information


Appendix S1.

Appendix S2.

Appendix S3.

Appendix S4.

Appendix S5.

Appendix S6.

## Data Availability

The data that support the findings of this study are openly available in Dryad Data Repository at https://doi.org/10.5061/dryad.1vhhmgr12.
