# Supplementary material for: A checklist of European butterfly larval foodplants
Source: Ecol Evol. 2024 Jan 7;14(1):e10834. doi: 10.1002/ece3.10834 (PMC10771928; doi:10.1002/ece3.10834)
Supplement: Supplementary file 3 — Appendix S3. [file ECE3-14-e10834-s006.pdf]

1 Checklist of European Papilionoidea

This checklist of European butterflies is based on Wiemers et al. (2018), with additions based on latest research (detailed below), and butterflies from the European part of Russia (Ilyina and Morgun, 2010, 2011; Kuznetsov, 2009, 2011; Mamedova, 2014). Changes to Wiemers et al. (2018) are highlighted in blue. The list is ordered according to the complete time-calibrated multi-gene phylogeny of the European butterflies (Wiemers et al., 2020), with additions placed in their approximate place.

Papilio demoleus is a recent colonisation of Cyprus (John et al., 2022; John and Makris, 2022). *Papilio saharae* was discovered breeding on the Italian island of Lampedusa (Cassar and Catania, 2022; Cassar et al., 2023).

The DNA analysis of all the European butterflies shows that the genus ***Carcharodus*** is split into two clades (Wiemers et al., 2020). Examination of genital structure showed that the genus *Carcharodus* should be split into the genera *Carcharodus* and *Reverdinus* (Coutsis, 2016). This split is supported by the DNA analysis of the subtribe *Carcharodina* of the tribe *Carcharodini* which all European species belong to (Zhang et al., 2020). That analysis removed *Muschampia cribrellum* from the genus *Muschampia* placing into the genus *Favria*, and placed all members of the genus *Reverdinus* at the subgenus level in the genus *Muschampia*, with existing European members of the genus belonging to the *Muschampia*. Two new species have been added following the split of *Muschampia proto* by Hinojosa et al. (2021).

The genus ***Euchloe*** as defined in the latest European Checklist (Wiemers et al., 2018) is not monophyletic. Back (2020) has divided the genus into three which is consistent with the phylogeny published by Wiemers et al. (2020). As there are also clear morthological differences between the three genera, the Portuguese Dappled White and the Greenish Black-tips have been renamed to follow Back (2020).

The distribution of the genus *Zegris* is disjointed, with a western population in Spain and Algaria, which Back (2012, 2020) considers is the species *Z. meridionalis*, whereas Wiemers et al. (2018) considers it a subspecies of *Z. eupheme*, which along with the other three species in *Zegris* have an eastern distribution from southern Ukraine eastwards. Given the disjointed distribution, along with the DNA evidence and morphological differences, it seems best to treat *Z. meridionalis* and *Z. eupheme* as separate species.

Recent DNA analysis suggests that the genus *Cyclyrius* should be considered a junior synonym of *Leptotes* (Fric et al., 2019).

Melitaea ornata has been split into two species, with *Melitaea pseudornata* present in the Iberian Peninnisula, and *Melitaea ornata* distributed in the rest of Europe (Sánchez Mesa and Muñoz Sairot, 2017; Muñoz Sairot and Sánchez Mesa, 2019).

In a review of the genus *Hyponephele* (Lukhtanov and Pazhenkova, 2021) added *Hyponephele mauritanica* to the Iberian Peninsula, which occurs sympatrically with *Hyponephele lycaon*, but differs morphologicaly. Other European species of *Hyponephele* are allopatric in their distribution.

In a review of the taxonomic name *Papilio hermione* Linnaeus (Russell and Vane-Wright, 2022), and the confusion in the scientific names for the Woodland Grayling *Hipparchia fagi* Scopoli, 1763 and the Rock Grayling *Hipparchia alcyone* [Denis & Schiffermüller], 1775, the scientific name for the Rock Grayling has been changed to *Hipparchia alcyone* (Denis & Schiffermüller, 1775).

Papilionidae Latreille, 1802

Parnassiinae Duponchel, [1835]

Parnassiini Duponchel, [1835]

Parnassius Latreille, 1804

Parnassius nordmanni (Ménétriés, 1850)	
Parnassius mnemosyne (Linnaeus, 1758)	Clouded Apollo
Parnassius apollo (Linnaeus, 1758)	Apollo
Parnassius phoebus (Fabricius, 1793)	Small Apollo

Luehdorfiini Chapman, 1895

Archon Hübner, 1822

Archon apollinus (Herbst, 1798)	False Apollo

Zerynthiini Grote, 1899

Zerynthia Ochsenheimer, 1816

Zerynthia rumina (Linnaeus, 1758)	Spanish Festoon
Zerynthia cassandra (Geyer, [1828])	Italian Festoon
Zerynthia polyxena ([Denis & Schiffermüller], 1775)	Southern Festoon
Zerynthia cretica (Rebel, 1904)	Cretan Festoon
Zerynthia cerisy (Godart, [1824])	Eastern Festoon
Zerynthia caucasica (Lederer, 1864)	

Papilioninae Latreille, [1802]

Papilionini Latreille, [1802]	
Iphiclides Hübner, 1819	
Iphiclides feisthamelii (Duponchel, 1832)	Iberian Scarce Swallowtail
Iphiclides podalirius (Linnaeus, 1758)	Scarce Swallowtail
Papilio Linnaeus, 1758	
Papilio alexanor Esper, 1800	Southern Swallowtail
Papilio hospiton Gené, 1839	Corsican Swallowtail
Papilio machaon Linnaeus, 1758	Swallowtail
Papilio demoleus Linnaeus, 1758	Lime Swallowtail
Papilio saharae Oberthür, 1879	Sahara Swallowtail

Hesperiidae Latreille, 1809

Pyrginae Burmeister, 1878

Carcharodini Verity, 1940	
Carcharodus Hübner, 1819	
Carcharodus tripolinus (Verity, 1925)	False Mallow Skipper
Carcharodus alceae (Esper, 1780)	Mallow Skipper
Spialia Swinhoe, 1912	
Spialia phlomidis (Herrich-Schäffer, 1845)	Persian Skipper
Spialia therapne (Rambur, 1832)	Corsican Red-underwing Skipper
Spialia sertorius (Hoffmansegg, 1804)	Red-underwing Skipper
Spialia rosae Hernández-Roldán, Dapporto, Dincă, Vicente & Vila, 2016	
Spialia orbifer (Hübner, [1823])	Hungarian Skipper
Favria Tutt, 1906	
Favria cribrellum (Eversmann, 1841)	Spinose Skipper
Muschampia Tutt, 1906	
Muschampia tessellum (Hübner, [1803])	Tessellated Skipper
Muschampia proto (Ochsenheimer, 1808)	Sage Skipper
Muschampia alta (Schwingenschuss, 1942)	
Muschampia proteides (Wagner, 1929)	
Muschampia orientalis Reverdin, 1913	Oriental Marbled Skipper
Muschampia floccifera (Zeller, 1847)	Tufted Marbled Skipper
Muschampia lavatherae (Esper, 1783)	Marbled Skipper
Muschampia baeticus (Rambur, 1839)	Southern Marbled Skipper
Muschampia stauderi Reverdin, 1913	False Marbled Skipper
Erynnini Brues & Melander, 1932	
Erynnis Schrank, 1801	
Erynnis tages (Linnaeus, 1758)	Dingy Skipper
Erynnis marloyi (Boisduval, 1834)	Inky Skipper
Pyrgini Burmeister, 1878	
Pyrgus Hübner, 1819	
Pyrgus sidae (Esper, 1784)	Yellow-banded Skipper
Pyrgus carthami (Hübner, [1813])	Safflower Skipper
Pyrgus malvoides (Elwes & Edwards, 1897)	Southern Grizzled Skipper
Pyrgus malvae (Linnaeus, 1758)	Grizzled Skipper
Pyrgus melotis (Duponchel, [1834])	Aegean skipper
Pyrgus andromedae (Wallengren, 1853)	Alpine Grizzled Skipper
Pyrgus cacaliae (Rambur, 1839)	Dusky Grizzled Skipper
Pyrgus centaureae (Rambur, 1839)	Northern Grizzled Skipper
Pyrgus serratulae (Rambur, 1839)	Olive Skipper
Pyrgus armoricanus (Oberthür, 1910)	Oberthür's Grizzled Skipper
Pyrgus jupei Alberti, 1967	Caucasian skipper
Pyrgus cinarae (Rambur, 1839)	Sandy Grizzled Skipper
Pyrgus cirsii (Rambur, 1839)	Cinquefoil Skipper
Pyrgus carlinae (Rambur, 1839)	Carline Skipper
Pyrgus onopordi (Rambur, 1839)	Rosy Grizzled Skipper
Pyrgus warrenensis (Verity, 1928)	Warren's Skipper
Pyrgus foulquieri (Oberthür, 1910)	Foulquier's Grizzled Skipper
Pyrgus alveus (Hübner, [1803])	Large Grizzled Skipper

Heteropterinae Aurivillius, 1925

Heteropterus Duméril, 1806

Heteropterus morpheus (Pallas, 1771)	Large Chequered Skipper
Carterocephalus Lederer, 1852	
Carterocephalus silvicola (Meigen, 1829)	Northern Chequered Skipper
Carterocephalus palaemon (Pallas, 1771)	Chequered Skipper
**Hesperiinae** Latreille, 1809	
Baorini Doherty, 1866	
Borbo Evans, 1949	
Borbo borbonica (Boisduval, 1833)	Zeller's Skipper
Pelopidas Walker, 1870	
Pelopidas thrax (Hübner, [1821])	Millet Skipper
Gegenes Hübner, 1819	
Gegenes nostrodamus (Fabricius, 1793)	Mediterranean Skipper
Gegenes pumilio (Hoffmansegg, 1804)	Pygmy Skipper
Thymelicini Tutt 1905	
Thymelicus Hübner, 1819	
Thymelicus christi Rebel, 1894	Canarian Skipper
Thymelicus acteon (Rottemburg, 1775)	Lulworth Skipper
Thymelicus hyrax (Lederer, 1861)	Levantine Skipper
Thymelicus sylvestris (Poda, 1761)	Small Skipper
Thymelicus lineola (Ochsenheimer, 1808)	Essex Skipper
Hesperiini Latreille 1809	
Ochlodes Scudder, 1872	
Ochlodes sylvanus (Esper, 1777)	Large Skipper
Hesperia Fabricius, 1793	
Hesperia comma (Linnaeus, 1758)	Silver-spotted Skipper
**Pieridae** Swainson, 1820	
**Dismorphiinae** Schatz , 1887	
Leptideini Grote, 1897	
Leptidea Billberg, 1820	
Leptidea duponcheli (Staudinger, 1871)	Eastern Wood White
Leptidea morsei (Fenton, 1882)	Fenton's Wood White
Leptidea juvernica Williams, 1946	Cryptic Wood White
Leptidea sinapis (Linnaeus, 1758)	Wood White
Leptidea reali Reissinger, 1990	Réal's Wood White
**Coliadinae** Swainson, 1827	
Rhodocerini Duponchell, [1835]	
Gonepteryx Leach, 1815	
Gonepteryx rhamni (Linnaeus, 1758)	Brimstone
Gonepteryx cleobule (Hübner, [1831])	Canary Brimstone
Gonepteryx farinosa (Zeller, 1847)	Powdered Brimstone
Gonepteryx cleopatra (Linnaeus, 1767)	Cleopatra
Gonepteryx maderensis C. Felder, 1862	Madeiran Brimstone
Coliadini Swainson, 1827	
Catopsilia Hübner, 1819	
Catopsilia florella (Fabricius, 1775)	African Migrant
Colias Fabricius, 1807	
Colias hyale (Linnaeus, 1758)	Pale Clouded Yellow
Colias alfacariensis Ribbe, 1905	Berger's Clouded Yellow
Colias chrysotheme (Esper, [1781])	Lesser Clouded Yellow
Colias aurorina Herrich-Schäffer, 1850	Greek Clouded Yellow
Colias phicomone (Esper, [1780])	Mountain Clouded Yellow
Colias erate (Esper, [1805])	Eastern Pale Clouded Yellow
Colias crocea (Geoffroy, 1785)	Clouded Yellow
Colias caucasica Staudinger, 1871	Balkan Clouded Yellow
Colias thisoa Ménétriès, 1832	
Colias myrmidone (Esper, [1781])	Danube Clouded Yellow
Colias hecla Lefèbvre, 1836	Northern Clouded Yellow
Colias palaeno (Linnaeus, [1760])	Moorland Clouded Yellow
Colias tyche (Böber, 1812)	Pale Arctic Clouded Yellow
**Pierinae** Swainson, 1820	

Teracolini Reuter, 1896	
Colotis Hübner, 1819	
Colotis evagore (Klug, 1829)	Desert Orange-tip
Anthocharini Tutt, 1894	
Iberochloe Back, Knebelsberger & Miller, 2008	
Iberochloe tagis (Hübner, [1804])	Portuguese Dappled White
Elphinstonia Klots, 1930	
Elphinstonia charlonia (Donzel, 1842)	Greenish Black-tip
Elphinstonia bazae Fabiano, 1993	Spanish Greenish Black-tip
Elphinstonia penia (Freyer, 1851)	Eastern Greenish Black-tip
Anthocharis Boisduval, Rambur, Duméril & Graslin, 1833	
Anthocharis euphenoides Staudinger, 1869	Provence Orange-tip
Anthocharis cardamines (Linnaeus, 1758)	Orange-tip
Anthocharis damone Boisduval, 1836	Eastern Orange-tip
Anthocharis gruneri Herrich-Schäffer, 1851	Grüner's Orange-tip
Zegris Boisduval, 1836	
Zegris meridionalis (Lederer, 1853)	
Zegris eupheme (Esper, [1804])	Sooty Orange-tip
Zegris pyrothoe (Eversmann, 1832)	
Euchloe Hübner, 1819	
Euchloe eversi Stamm, 1963	
Euchloe grancanariensis Acosta, 2008	
Euchloe hesperidum Rothschild, 1913	Canary Green-striped White
Euchloe belemia (Esper, 1800)	Green-striped White
Euchloe ausonia (Hübner, [1804])	Eastern Dappled White
Euchloe simplonia (Freyer, 1829)	Mountain Dappled White
Euchloe insularis (Staudinger, 1861)	Corsican Dappled White
Euchloe crameri Butler, 1869	Western Dappled White
Pierini Duponchel, [1835]	
Aporiina Chapman, 1895	
Aporia Hübner, 1819	
Aporia crataegi (Linnaeus, 1758)	Black-veined White
Pierina Swainson, 1820	
Pontia Fabricius, 1807	
Pontia chloridice (Hübner, [1813])	Small Bath White
Pontia callidice (Hübner, [1800])	Peak White
Pontia edusa (Fabricius, 1777)	Eastern Bath White
Pontia daplidice (Linnaeus, 1758)	Bath White
Pieris Schrank, 1801	
Pieris brassicae (Linnaeus, 1758)	Large White
Pieris wollastoni (Butler, 1886)	Madeiran Large White
Pieris cheiranthi (Hübner, [1808])	Canary Islands Large White
Pieris krueperi Staudinger, 1860	Krueper's Small White
Pieris rapae (Linnaeus, 1758)	Small White
Pieris mannii (Mayer, 1851)	Southern Small White
Pieris ergane (Geyer, [1828])	Mountain Small White
Pieris bryoniae (Hübner, [1806])	Mountain Green-veined White
Pieris napi (Linnaeus, 1758)	Green-veined White
Pieris balcana Lorković, [1969]	Balkan Green-veined White
**Riodinidae** Grote, 1895	
**Nemeobiinae** Bates 1868	
Nemeobiini Bates 1868	
Hamearis Hübner, 1819	
Hamearis lucina (Linnaeus, 1758)	Duke of Burgundy
**Lycaenidae** Leach, 1815	
**Aphnaeinae** Distant 1884	
Cigaritis Moore, 1881	
Cigaritis acamas (Klug, 1834)	Levantine Leopard
**Lycaeninae** Leach, 1815	
Lycaenini Leach, 1815	

Lycaena Fabricius, 1807	
Lycaena dimorpha (Staudinger, 1881)	
Lycaena phlaeas (Linnaeus, [1760])	Small Copper
Lycaena bleusei (Oberthür, 1884)	Iberian Sooty Copper
Lycaena virgaureae (Linnaeus, 1758)	Scarce Copper
Lycaena ottomana (Lefèbvre, [1831])	Grecian Copper
Lycaena tityrus (Poda, 1761)	Sooty Copper
Lycaena helle ([Denis & Schiffermüller], 1775)	Violet Copper
Lycaena alciphron (Rottemburg, 1775)	Purple-shot Copper
Lycaena dispar ([Haworth], 1802)	Large Copper
Lycaena thetis Klug, 1834	Fiery Copper
Lycaena thersamon (Esper, 1784)	Lesser Fiery Copper
Lycaena hippothoe (Linnaeus, [1760])	Purple-edged Copper
Lycaena candens (Herrich-Schäffer, 1844)	Balkan Copper
**Theclinae** Swainson 1831	
Theclini Swainson 1831	
Laeosopis Rambur, 1858	
Laeosopis roboris (Esper, [1793])	Spanish Purple Hairstreak
Thecla Fabricius, 1807	
Thecla betulae (Linnaeus, 1758)	Brown Hairstreak
Favonius Sibatani & Ito, 1942	
Favonius quercus (Linnaeus, 1758)	Purple Hairstreak
Tomarini Eliot 1973	
Tomares Rambur, 1840	
Tomares ballus (Fabricius, 1787)	Provence Hairstreak
Tomares nogelii (Herrich-Schäffer, 1851)	Nogel's Hairstreak
Tomares callimachus (Eversmann, 1848)	
Eumaeini Doubleday 1847	
Callophryidina Tutt, 1907	
Callophrys Billberg, 1820	
Callophrys avis Chapman, 1909	Chapman's Green Hairstreak
Callophrys rubi (Linnaeus, 1758)	Green Hairstreak
Callophrys suaveola (Staudinger, 1881)	
Callophrys chalybeitincta Sovinsky, 1905	
Neolycaena de Niceville, 1890	
Neolycaena rhymnus (Eversmann, 1832)	
Satyrium Scudder, 1876	
Satyrium pruni (Linnaeus, 1758)	Black Hairstreak
Satyrium w-album (Knoch, 1782)	White-letter Hairstreak
Satyrium acaciae (Fabricius, 1787)	Sloe Hairstreak
Satyrium spini ([Denis & Schiffermüller], 1775)	Blue-spot Hairstreak
Satyrium ledereri (Boisduval, 1848)	Orange-banded Hairstreak
Satyrium ilicis (Esper, 1779)	Ilex Hairstreak
Satyrium esculi (Hübner, [1804])	False Ilex Hairstreak
**Polyommatinae** Swainson 1831	
Polyommatini Swainson 1831	
Azanina	
Azanus Moore, 1881	
Azanus ubaldus (Stoll, 1782)	Bright Babul Blue
Azanus jesous (Guérin-Méneville, 1849)	African Babul Blue
Zizeeriina	
Zizeeria Chapman, 1910	
Zizeeria knysna (Trimen, 1862)	African Grass Blue
Zizeeria karsandra (Moore, 1865)	Dark Grass Blue
Castaliina	
Tarucus Moore, 1881	
Tarucus theophrastus (Fabricius, 1793)	Common Tiger Blue
Tarucus balkanicus (Freyer, 1844)	Little Tiger Blue
Cacyreina	
Cacyreus Butler, 1898	
Cacyreus marshalli Butler, 1898	Geranium Bronze

Lampidina	
Lampides Hübner, 1819	
Lampides boeticus (Linnaeus, 1767)	Long-tailed Blue
Lycaenopsina	
Celastrina Tutt, 1906	
Celastrina argiolus (Linnaeus, 1758)	Holly Blue
Scolitantidina Hübner, [1819]	
Phengaris Doherty, 1891	
Phengaris alcon ([Denis & Schiffermüller], 1775)	Alcon Blue
Phengaris arion (Linnaeus, 1758)	Large Blue
Phengaris teleius (Bergsträsser, 1779)	Scarce Large Blue
Phengaris nausithous (Bergsträsser, 1779)	Dusky Large Blue
Scolitantides Hübner, 1819	
Scolitantides orion (Pallas, 1771)	Chequered Blue
Praephilotes Forster, 1938	
Praephilotes anthracias (Christoph, 1877)	
Iolana Bethune-Baker, 1914	
Iolana iolas (Ochsenheimer, 1816)	Iolas Blue
Iolana debilitata (Schultz, 1905)	
Glaucopsyche Scudder, 1872	
Glaucopsyche melanops (Boisduval, 1828)	Black-eyed Blue
Glaucopsyche alexis (Poda, 1761)	Green-underside Blue
Glaucopsyche paphos Chapman, 1920	Paphos Blue
Turanana Bethune-Baker, 1916	
Turanana taygetica (Rebel, 1902)	Odd-spot Blue
Pseudophilotes Beuret, 1958	
Pseudophilotes bavius (Eversmann, 1832)	Bavius Blue
Pseudophilotes barbagiae De Prins & van der Poorten, 1982	Sardinian Blue
Pseudophilotes abencerragus (Pierret, 1837)	False Baton Blue
Pseudophilotes panoptes (Hübner, [1813])	Panoptes Blue
Pseudophilotes baton (Bergsträsser, 1779)	Baton Blue
Pseudophilotes vicrama (Moore, 1865)	Eastern Baton Blue
Leptotina	
Leptotes Scudder, 1876	
Leptotes webbianus (Brullé, 1839)	Canary Blue
Leptotes pirithous (Linnaeus, 1767)	Lang's Short-tailed Blue
Everina	
Tongeia Tutt, 1908	
Tongeia fischeri (Eversmann, 1843)	
Cupido Schrank, 1801	
Cupido argiades (Pallas, 1771)	Short-tailed Blue
Cupido decoloratus (Staudinger, 1886)	Eastern Short-tailed Blue
Cupido alcetas (Hoffmansegg, 1804)	Provençal Short-tailed Blue
Cupido osiris (Meigen, 1829)	Osiris Blue
Cupido minimus (Fuessly, 1775)	Small Blue
Cupido lorquinii (Herrich-Schäffer, 1850)	Lorquin's Blue
Polyommatina Swainson 1831	
Luthrodes Moore, 1893	
Luthrodes galba (Lederer, 1855)	Small Desert Blue
Freyeria Sibatani & Ito, 1942	
Freyeria trochylus (Freyer, 1844)	Grass Jewel
Plebejus Kluk, 1780	
Plebejus argus (Linnaeus, 1758)	Silver-studded Blue
Plebejus idas (Linnaeus, [1760])	Idas Blue
Plebejus bellieri (Oberthür, 1910)	Bellier's Blue
Plebejus argyrognomon (Bergsträsser, 1779)	Reverdin's Blue
Plebejus maracandicus (Erschoff, 1874)	
Plebejidea Schrank, 1801	
Plebejidea loewii (Zeller, 1847)	Loew's Blue
Eumedonia Hübner, 1819	
Eumedonia eumedon (Esper, 1780)	Geranium Argus

Kretania Moore, 1893	
Kretania eurypilus (Freyer, 1851)	Eastern Brown Argus
Kretania hesperica (Rambur, 1839)	Spanish Zephyr Blue
Kretania trappi (Verity, 1927)	Alpine Zephyr Blue
Kretania sephirus (Frivaldszky, 1835)	Zephyr Blue
Kretania pylaon (Fischer, 1832)	
Kretania zephyrinus (Erschoff, 1874)	
Cyaniris Dalman, 1816	
Cyaniris semiargus (Rottemburg, 1775)	Mazarine Blue
Agriades Dalman, 1816	
Agriades optilete (Knoch, 1781)	Cranberry Blue
Kretania Moore, 1893	
Kretania psylorita (Freyer, 1845)	Cretan Argus
Agriades Dalman, 1816	
Agriades orbitulus (Prunner, 1798)	Alpine Blue
Agriades dardanus (Freyer, 1843)	Bosnian blue
Agriades pyrenaicus (Boisduval, 1840)	Gavarnie Blue
Agriades zullichi Hemming, 1933	Zullich´s Blue
Agriades aquilo (Boisduval, 1832)	Arctic Blue
Agriades glandon (Prunner, 1798)	Glandon Blue
Glabroculus Hübner, 1819	
Glabroculus cyane (Eversmann, 1837)	
Aricia Reichenbach, 1817	
Aricia morronensis (Ribbe, 1910)	Spanish Argus
Aricia anteros (Freyer, 1838)	Blue Argus
Aricia cramera (Eschscholtz, 1821)	Southern Brown Argus
Aricia nicias (Meigen, 1829)	Silvery Argus
Aricia artaxerxes (Fabricius, 1793)	Northern Brown Argus
Aricia montensis Verity, 1928	Southern Mountain Argus
Aricia agestis ([Denis & Schiffermüller], 1775)	Brown Argus
Aricia teberdina (Sheljuzhko, 1934)	
Neolysandra de Niceville, 1890	
Neolysandra coelestina (Eversmann, 1843)	Pontic Blue
Lysandra Fabricius, 1807	
Lysandra bellargus (Rottemburg, 1775)	Adonis Blue
Lysandra corydonius (Herrich-Schäffer, 1852)	
Lysandra coridon (Poda, 1761)	Chalkhill Blue
Lysandra caelestissima (Verity, 1921)	Azure Chalkhill Blue
Lysandra hispana (Herrich-Schäffer, 1851)	Provence Chalkhill Blue
Lysandra albicans (Gerhard, 1851)	Spanish Chalkhill Blue
Polyommatus Latreille, 1804	
Polyommatus escheri (Hübner, [1823])	Escher's Blue
Polyommatus thersites (Cantener, 1835)	Chapman's Blue
Polyommatus amandus (Schneider, 1792)	Amanda's Blue
Polyommatus daphnis ([Denis & Schiffermüller], 1775)	Meleager's Blue
Polyommatus icarus (Rottemburg, 1775)	Common Blue
Polyommatus eros (Ochsenheimer, 1808)	Eros Blue
Polyommatus celina (Austaut, 1879)	Southern Common Blue
Polyommatus nivescens (Keferstein, 1851)	Mother-of-Pearl Blue
Polyommatus dorylas ([Denis & Schiffermüller], 1775)	Turquoise Blue
Polyommatus golgus (Hübner, [1813])	Nevada Blue
Polyommatus damon ([Denis & Schiffermüller], 1775)	Damon Blue
Polyommatus iphigenia (Herrich-Schäffer, 1847)	Chelmos Blue
Polyommatus damocles (Herrich-Schäffer, 1844)	
Polyommatus admetus (Esper, 1783)	Anomalous Blue
Polyommatus nephohiptamenos (Brown & Coutsis, 1978)	Higgins' Anomalous Blue
Polyommatus ripartii (Freyer, 1830)	Ripart's Anomalous Blue
Polyommatus damone (Eversmann, 1841)	
Polyommatus violetae (Gómez-Bustillo, Expósito & Martínez, 1979)	Andalusian Anomalous Blue
Polyommatus fulgens (Sagarra, 1925)	Catalonian Furry Blue
Polyommatus fabressei (Oberthür, 1910)	Oberthür's Anomalous Blue

Polyommatus dolus (Hübner, [1823])	Furry Blue
Polyommatus humedasae (Toso & Balletto, 1976)	Piedmont Anomalous Blue
Polyommatus orphicus Kolev, 2005	Kolev's Anomalous Blue
Polyommatus aroaniensis (Brown, 1976)	Grecian Anomalous Blue
Polyommatus timfristos Lukhtanov, Vishnevskaya & Shapoval, 2016	
Polyommatus yurinekrutenko Koçak, 1996	
Polyommatus phyllis (Staudinger, 1886)	
Polyommatus aserbeidschanus Forster, 1956	
Polyommatus shamil (Dantchenko, 2000)	

Nymphalidae Rafinesque, 1815

Limenitidinae Behr, 1864

Neptini Newman, 1870	
Neptis Fabricius, 1807	
Neptis rivularis (Scopoli, 1763)	Hungarian Glider
Neptis sappho (Pallas, 1771)	Common Glider
Limenitidini Behr, 1864	
Limenitis Fabricius, 1807	
Limenitis populi (Linnaeus, 1758)	Poplar Admiral
Limenitis reducta Staudinger, 1901	Southern White Admiral
Limenitis camilla (Linnaeus, 1764)	White Admiral

Heliconiinae Swainson, 1822

Argynnini Duponchel, 1835	
Issoria Hübner, 1819	
Issoria eugenia (Eversmann, 1847)	
Issoria lathonia (Linnaeus, 1758)	Queen of Spain Fritillary
Brenthis Hübner, 1819	
Brenthis hecate ([Denis & Schiffermüller], 1775)	Twin-spot Fritillary
Brenthis daphne ([Denis & Schiffermüller], 1775)	Marbled Fritillary
Brenthis ino (Rottemburg, 1775)	Lesser Marbled Fritillary
Argynnis Fabricius, 1807	
Argynnis pandora ([Denis & Schiffermüller], 1775)	Cardinal
Argynnis paphia (Linnaeus, 1758)	Silver-washed Fritillary
Argynnis laodice (Pallas, 1771)	Pallas' Fritillary
Speyeria Scudder, 1872	
Speyeria aglaja (Linnaeus, 1758)	Dark Green Fritillary
Fabriciana Reuss, 1920	
Fabriciana adippe ([Denis & Schiffermüller], 1775)	High Brown Fritillary
Fabriciana elisa (Godart, 1823)	Corsican Fritillary
Fabriciana niobe (Linnaeus, 1758)	Niobe Fritillary
Boloria Moore, 1900	
Boloria eunomia (Esper, 1800)	Bog Fritillary
Boloria caucasica (Lederer, 1852)	
Boloria graeca (Staudinger, 1870)	Balkan Fritillary
Boloria pales ([Denis & Schiffermüller], 1775)	Shepherd's Fritillary
Boloria napaea (Hoffmansegg, 1804)	Mountain Fritillary
Boloria aquilonaris (Stichel, 1908)	Cranberry Fritillary
Boloria alaskensis (Holland, 1900)	
Boloria polaris (Boisduval, 1828)	Polar Fritillary
Boloria tritonia (Böber, 1812)	
Boloria frigga (Thunberg, 1791)	Frigga's Fritillary
Boloria improba (Butler, 1877)	Dusky-winged Fritillary
Boloria selene ([Denis & Schiffermüller], 1775)	Small Pearl-bordered Fritillary
Boloria thore (Hübner, [1804])	Thor's Fritillary
Boloria dia (Linnaeus, 1767)	Weaver's Fritillary
Boloria freija (Thunberg, 1791)	Freija's Fritillary
Boloria euphrosyne (Linnaeus, 1758)	Pearl-bordered Fritillary
Boloria oscarus (Eversmann, 1844)	
Boloria selenis (Eversmann, 1837)	
Boloria titania (Esper, [1793])	Titania's Fritillary
Boloria chariclea (Schneider, 1794)	Arctic Fritillary

Boloria angarensis (Erschoff, 1870)	
**Apaturinae** Boisduval, 1840	
Apaturini Boisduval, 1840	
Apatura Fabricius, 1807	
Apatura iris (Linnaeus, 1758)	Purple Emperor
Apatura metis Freyer, 1829	Freyer's Purple Emperor
Apatura ilia ([Denis & Schiffermüller], 1775)	Lesser Purple Emperor
Thaleropis Staudinger 1871	
Thaleropis ionia (Fischer de Waldheim & Eversmann, 1851)	
**Nymphalinae** Rafinesque, 1815	
Nymphalini Rafinesque, 1815	
Araschnia Hübner, 1819	
Araschnia levana (Linnaeus, 1758)	Map
Vanessa Fabricius, 1807	
Vanessa cardui (Linnaeus, 1758)	Painted Lady
Vanessa virginiensis (Drury, 1773)	American Painted Lady
Vanessa vulcania Godart, 1819	Canary Red Admiral
Vanessa atalanta (Linnaeus, 1758)	Red Admiral
Aglais Dalman, 1816	
Aglais io (Linnaeus, 1758)	Peacock
Aglais urticae (Linnaeus, 1758)	Small Tortoiseshell
Aglais ichnusa (Hübner, [1824])	Corsican/Sardinian Small Tortoiseshell
Polygonia Hübner, 1819	
Polygonia egea (Cramer, 1775)	Southern Comma
Polygonia c-album (Linnaeus, 1758)	Comma
Nymphalis Kluk, 1780	
Nymphalis vaualbum ([Denis & Schiffermüller], 1775)	False Comma
Nymphalis polychloros (Linnaeus, 1758)	Large Tortoiseshell
Nymphalis xanthomelas ([Denis & Schiffermüller], 1775)	Yellow-legged Tortoiseshell
Nymphalis antiopa (Linnaeus, 1758)	Camberwell Beauty
Junoniini Reuter,1896	
Hypolimnas Hübner, 1819	
Hypolimnas misippus (Linnaeus, 1764)	Danaid Eggfly
Melitaeini Newman, 1870	
Euphydryina Higgins, 1978	
Euphydryas Scudder, 1872	
Euphydryas desfontainii (Godart, 1819)	Spanish Fritillary
Euphydryas aurinia (Rottemburg, 1775)	Marsh Fritillary
Euphydryas cynthia ([Denis & Schiffermüller], 1775)	Cynthia's Fritillary
Euphydryas iduna (Dalman, 1816)	Lapland Fritillary
Euphydryas intermedia (Ménétriés, 1859)	Asian Fritillary
Euphydryas maturna (Linnaeus, 1758)	Scarce Fritillary
Melitaeina Newman, 1870	
Melitaea Fabricius, 1807	
Melitaea didyma (Esper, 1778)	Spotted Fritillary
Melitaea trivia ([Denis & Schiffermüller], 1775)	Lesser Spotted Fritillary
Melitaea arduinna (Esper, 1783)	Freyer's Fritillary
Melitaea aetherie (Hübner, [1826])	Aetherie Fritillary
Melitaea ornata Christoph, 1893	Eastern Knapweed Fritillary
Melitaea pseudornata Muñoz Sariot & Sánchez Mesa, 2019	
Melitaea phoebe ([Denis & Schiffermüller], 1775)	Knapweed Fritillary
Melitaea cinxia (Linnaeus, 1758)	Glanville Fritillary
Melitaea diamina (Lang, 1789)	False Heath Fritillary
Melitaea asteria Freyer, 1828	Little Fritillary
Melitaea aurelia Nickerl, 1850	Nickerl's Fritillary
Melitaea varia Herrich-Schäffer, 1851	Grisons Fritillary
Melitaea parthenoides Keferstein, 1851	Meadow Fritillary
Melitaea deione (Geyer, [1832])	Provençal Fritillary
Melitaea celadussa Fruhstorfer, 1910	Southern Heath Fritillary
Melitaea athalia (Rottemburg, 1775)	Heath Fritillary
Melitaea britomartis Assmann, 1847	Assmann's Fritillary

Melitaea persea Kollar, [1850]	
Melitaea caucasogenita Verity, 1930	
Melitaea robertsi Butler, 1880)	
**Libytheinae** Boisduval, 1833	
Libythea Fabricius, 1807	
Libythea celtis (Laicharting, 1782)	Nettle-tree Butterfly
**Danainae** Boisduval 1833	
Danaini Boisduval 1833	
Danaus Kluk, 1780	
Danaus plexippus (Linnaeus, 1758)	Monarch
Danaus chrysippus (Linnaeus, 1758)	Plain Tiger
**Charaxinae** Doherty, 1886	
Charaxini Guenée, 1865	
Charaxes Ochsenheimer, 1816	
Charaxes jasius (Linnaeus, 1767)	Two-tailed Pasha
**Satyrinae** Boisduval, 1833	
Satyrini Boisduval, 1833	
Parargina Tutt, 1896	
Lasiommata Westwood, 1841	
Lasiommata maera (Linnaeus, 1758)	Large Wall Brown
Lasiommata deidamia (Eversmann, 1851)	
Lasiommata petropolitana (Fabricius, 1787)	Northern Wall Brown
Lasiommata paramegaera (Hübner, [1824])	Corsican Wall Brown
Lasiommata megera (Linnaeus, 1767)	Wall Brown
Pararge Hübner, 1819	
Pararge xiphia (Fabricius, 1775)	Madeiran Speckled Wood
Pararge xiphioides Staudinger, 1871	Canary Speckled Wood
Pararge aegeria (Linnaeus, 1758)	Speckled Wood
Kirinia Moore, 1893	
Kirinia climene (Esper, 1783)	Lesser Lattice Brown
Kirinia roxelana (Cramer, 1777)	Lattice Brown
Lopinga Moore, 1893	
Lopinga achine (Scopoli, 1763)	Woodland Brown
Coenonymphina Tutt 1896	
Coenonympha Hübner, 1819	
Coenonympha phryne (Pallas, 1771)	
Coenonympha oedippus (Fabricius, 1787)	False Ringlet
Coenonympha glycerion (Borkhausen, 1788)	Chestnut Heath
Coenonympha thyrsis (Freyer, 1845)	Cretan Small Heath
Coenonympha pamphilus (Linnaeus, 1758)	Small Heath
Coenonympha tullia (Müller, 1764)	Large Heath
Coenonympha amaryllis (Stoll, 1782)	
Coenonympha rhodopensis Elwes, 1900	Eastern Large Heath
Coenonympha dorus (Esper, 1782)	Dusky Heath
Coenonympha corinna (Hübner, [1804])	Corsican Heath
Coenonympha hero (Linnaeus, [1760])	Scarce Heath
Coenonympha leander (Esper, 1784)	Russian Heath
Coenonympha gardetta (Prunner, 1798)	Alpine Heath
Coenonympha arcania (Linnaeus, [1760])	Pearly Heath
Coenonympha orientalis Rebel, 1909	Balkan Heath
Subtribe ?	
Proterebia Roos & Arnscheid, 1980	
Proterebia phegea (Borkhausen, 1788)	Dalmatian Ringlet
Ypthimina Reuter, 1896	
Ypthima Hübner, 1818	
Ypthima asterope (Klug, 1832)	African Ringlet
Melanargiina Wheeler, 1903	
Melanargia Meigen, 1828	
Melanargia arge (Sulzer, 1776)	Italian Marbled White
Melanargia occitanica (Esper, [1793])	Western Marbled White
Melanargia pherusa (Boisduval, 1833)	Sicilian Marbled White

Melanargia ines (Hoffmansegg, 1804)	Spanish Marbled White
Melanargia russiae (Esper, 1783)	Esper's Marbled White
Melanargia larissa (Geyer, [1828])	Balkan Marbled White
Melanargia lachesis (Hübner, 1790)	Iberian Marbled White
Melanargia galathea (Linnaeus, 1758)	Marbled White
Satyrina Boisduval, 1833	
Hipparchia Fabricius, 1807	
Hipparchia statilinus (Hufnagel, 1766)	Tree Grayling
Hipparchia fatua Freyer, 1843	Freyer's Grayling
Hipparchia tamadabae Owen & Smith, 1992	
Hipparchia wyssii (Christ, 1889)	Canary Grayling
Hipparchia tilosi Manil, 1984	
Hipparchia bacchus (Higgins, 1967)	
Hipparchia fidia (Linnaeus, 1767)	Striped Grayling
Hipparchia gomera (Higgins, 1967)	
Hipparchia neomiris (Godart, 1823)	Corsican Grayling
Hipparchia fagi (Scopoli, 1763)	Woodland Grayling
Hipparchia syriaca (Staudinger, 1871)	Eastern Rock Grayling
Hipparchia autonoe (Esper, 1783)	
Hipparchia alcyone (Denis & Schiffermüller, 1775)	Rock Grayling
Hipparchia mersina (Staudinger, 1871)	Samos Grayling
Hipparchia miguelensis (Le Cerf, 1935)	Le Cerf's Grayling
Hipparchia azorina (Strecker, 1899)	Azores Grayling
Hipparchia maderensis (Bethune-Baker, 1891)	Madeiran Grayling
Hipparchia senthes (Fruhstorfer, 1908)	Balkan Grayling
Hipparchia aristaeus (Bonelli, 1826)	Southern Grayling
Hipparchia neapolitana (Stauder, 1921)	Italian Grayling
Hipparchia blachieri (Fruhstorfer, 1908)	Sicilian Grayling
Hipparchia volgensis (Mazokhin-Porshnyakov, 1952)	Delattin's Grayling
Hipparchia semele (Linnaeus, 1758)	Grayling
Hipparchia leighebi Kudrna, 1976	Eolian Grayling
Hipparchia pellucida (Stauder, 1924)	Lesbos Grayling
Hipparchia cretica (Rebel, 1916)	Cretan Grayling
Hipparchia christenseni Kudrna, 1977	Karpathos Grayling
Hipparchia cypriensis (Holik, 1949)	Cyprus Grayling
Hipparchia sbordonii Kudrna, 1984	Ponza Grayling
Oeneis Hübner, 1819	
Oeneis tarpeia (Pallas, 1771)	
Oeneis bore (Schneider, 1792)	Arctic Grayling
Oeneis ammon Elwes, 1899	
Oeneis melissa (Fabricius, 1775)	
Oeneis jutta (Hübner, [1806])	Baltic Grayling
Oeneis magna Graeser, 1888	
Oeneis norna (Thunberg, 1791)	Norse Grayling
Oeneis polixenes (Fabricius, 1775)	
Oeneis glacialis (Moll, 1785)	Alpine Grayling
Arethusana De Lesse, 1951	
Arethusana arethusa ([Denis & Schiffermüller], 1775)	False Grayling
Minois Hübner, 1819	
Minois dryas (Scopoli, 1763)	Dryad
Brintesia Frühstorfer, 1911	
Brintesia circe (Fabricius, 1775)	Great Banded Grayling
Satyrus Latreille, 1810	
Satyrus ferula (Fabricius, 1793)	Great Sooty Satyr
Satyrus actaea (Esper, 1781)	Black Satyr
Satyrus virbius Herrich-Schäffer, 1844	
Chazara Moore, 1893	
Chazara briseis (Linnaeus, 1764)	The Hermit
Chazara persephone (Hübner, [1805])	
Chazara prieuri (Pierret, 1837)	Southern Hermit
Pseudochazara De Lesse, 1951	

Pseudochazara graeca (Staudinger, 1870)	Grecian Grayling
Pseudochazara amymone Brown, 1976	Brown's Grayling
Pseudochazara geyeri (Herrich-Schäffer, 1846)	Grey Asian Grayling
Pseudochazara alpina (Staudinger, 1878)	
Pseudochazara anthelea (Hübner, [1824])	White-banded Grayling
Pseudochazara amalthea (Frivaldszky, 1845)	
Pseudochazara cingovskii (Gross, 1973)	Macedonian Grayling
Pseudochazara orestes De Prins & van der Poorten, 1981	Dils' Grayling
Pseudochazara tisiphone Brown, [1980]	Dark Grayling
Pseudochazara euxina (Kuznetsov, 1909)	
Pseudochazara williamsi (Romei, 1927)	Nevada Grayling
Pseudochazara mercurius (Staudinger, 1887)	
Pseudochazara nukatli Bogdanov, 2000	
Pseudochazara pelopea (Klug, 1832)	
Maniolina Grote, 1897	
Hyponephele Muschamp, 1915	
Hyponephele huebneri Koçak, 1980	
Hyponephele lycaon (Kühn, 1774)	Dusky Meadow Brown
Hyponephele lupina (Costa, 1836)	Oriental Meadow Brown
Hyponephele mauritanica ((Oberthür, 1881))	
Pyronia Hübner, 1819	
Pyronia bathseba (Fabricius, 1793)	Spanish Gatekeeper
Aphantopus Wallengren, 1853	
Aphantopus hyperantus (Linnaeus, 1758)	Ringlet
Pyronia Hübner, 1819	
Pyronia tithonus (Linnaeus, 1771)	Gatekeeper
Pyronia cecilia (Vallantin, 1894)	Southern Gatekeeper
Maniola Schrank, 1801	
Maniola cypricola (Graves, 1928)	Cyprus Meadow Brown
Maniola halicarnassus Thomson, 1990	Thomson's Meadow Brown
Maniola telmessia (Zeller, 1847)	Aegean Meadow Brown
Maniola jurtina (Linnaeus, 1758)	Meadow Brown
Maniola chia Thomson, 1987	Chios Meadow Brown
Maniola nurag (Ghiliani, 1852)	Sardinian Meadow Brown
Maniola megala (Oberthür, 1909)	Turkish Meadow Brown
Erebiina Tutt, 1896	
Erebia Dalman, 1816	
Erebia edda Ménétriés, 1851	
Erebia fasciata Butler, 1868	
Erebia discoidalis (Kirby, 1837)	
Erebia rossii (Curtis, 1835)	
Erebia cyclopius (Eversmann, 1844)	
Erebia embla (Thunberg, 1791)	Lapland Ringlet
Erebia disa (Thunberg, 1791)	Arctic Ringlet
Erebia epistygne (Hübner, [1819])	Spring Ringlet
Erebia ottomana Herrich-Schäffer, 1847	Ottoman Brassy Ringlet
Erebia callias Edwards, 1871	
Erebia iranica Grum-Grshimailo, 1895	
Erebia rondoui Oberthür, 1908	Pyrenees Brassy Ringlet
Erebia hispania Butler, 1868	Spanish Brassy Ringlet
Erebia nivalis Lorković & Lesse, 1954	De Lesse's Brassy Ringlet
Erebia calcarius Lorković, 1953	Lorkovic's Brassy Ringlet
Erebia tyndarus (Esper, 1781)	Swiss Brassy Ringlet
Erebia arvernensis Oberthür, 1908	Western Brassy Ringlet
Erebia cassioides (Hohenwarth, 1792)	Common Brassy Ringlet
Erebia neleus (Freyer, 1832)	
Erebia claudina (Borkhausen, 1789)	White Speck Ringlet
Erebia christi Rätzer, 1890	Rätzer's Ringlet
Erebia pharte (Hübner, [1804])	Blind Ringlet
Erebia epiphron (Knoch, 1783)	Mountain Ringlet
Erebia orientalis Elwes, 1900	Bulgarian Ringlet

Erebia jeniseiensis Trybom, 1877	
Erebia dabanensis Erschoff, 1872	
Erebia meolans (Prunner, 1798)	Piedmont Ringlet
Erebia palarica Chapman, 1905	Chapman's Ringlet
Erebia albergana (Prunner, 1798)	Almond-eyed Ringlet
Erebia triarius (Prunner, 1798)	de Prunner's Ringlet
Erebia polaris Staudinger, 1861	Arctic Woodland Ringlet
Erebia medusa ([Denis & Schiffermüller], 1775)	Woodland Ringlet
Erebia aethiops (Esper, 1777)	Scotch Argus
Erebia pandrose (Borkhausen, 1788)	Dewy Ringlet
Erebia sthennyo Graslin, 1850	False Dewy Ringlet
Erebia flavofasciata Heyne, 1895	Yellow-banded Ringlet
Erebia gorge (Hübner, [1804])	Silky Ringlet
Erebia pluto (Prunner, 1798)	Sooty Ringlet
Erebia sudetica Staudinger, 1861	Sudeten Ringlet
Erebia melampus (Fuessly, 1775)	Lesser Mountain Ringlet
Erebia manto ([Denis & Schiffermüller], 1775)	Yellow-spotted Ringlet
Erebia eriphyle (Freyer, 1836)	Eriphyle Ringlet
Erebia euryale (Esper, 1805)	Large Ringlet
Erebia ligea (Linnaeus, 1758)	Arran Brown
Erebia oeme (Hübner, [1804])	Bright-eyed Ringlet
Erebia gorgone Boisduval, 1833	Gavarnie Ringlet
Erebia aethiopellus (Hoffmansegg, 1806)	False Mnestra Ringlet
Erebia mnestra (Hübner, [1804])	Mnestra's Ringlet
Erebia rhodopensis Nicholl, 1900	Nicholl's Ringlet
Erebia scipio Boisduval, 1833	Larche Ringlet
Erebia melas (Herbst, 1796)	Black Ringlet
Erebia pronoe (Esper, 1780)	Water Ringlet
Erebia lefebvrei (Boisduval, 1828)	Lefèbvre's Ringlet
Erebia zapateri Oberthür, 1875	Zapater's Ringlet
Erebia neoridas (Boisduval, 1828)	Autumn Ringlet
Erebia montana (Prunner, 1798)	Marbled Ringlet
Erebia stiria (Godart, [1824])	Styrian Ringlet
Erebia styx (Freyer, 1834)	Stygian Ringlet

References

Back, W. (2012). Phaenotypische und genotypische abgrenzung der arten und unterarten der Gattung Zegris Boisduval, 1836 (Lepidoptera, Pieridae). *Atalanta*, 43(1/2):77–86.

Back, W. (2020). *Pieridae part IV: Subfamily Pierinae partim), Tribe Anthocharidini.* Guide to the butterflies of the Palearctic Region. Omnes Artes, Milano.

Cassar, L. F. and Catania, A. (2022). Preliminary findings on the presence of a taxon with morphological traits of Papilio saharae Oberthür, 1879 in Lampedusa (Italy) (Lepidoptera: Papilionidae). *SHILAP Revista de lepidopterologı ´a*, 50(198):303–312.

Cassar, L.-F., Catania, A., and Cotton, A. M. (2023). A new subspecies of Papilio saharae Oberthür, 1879 (Lepidoptera: Papilionidae) from Lampedusa, Italy. *Zootaxa*, 5231(1):65–78.

Coutsis, J. G. (2016). The male and female genital structures of skippers currently placed in the genus Carcharodus Hübner, [1819] and their taxonomic significance (Lepidoptera: Hesperiidae, Pyrginae). *Phegea*, 44.

Fric, Z. F., Maresova, J., Kadlec, T., Tropek, R., Pyrcz, T. W., and Wiemers, M. (2019). World travellers: phylogeny and biogeography of the butterfly genus Leptotes (Lepidoptera: Lycaenidae). *Systematic Entomology*, 44(3):652–665.

Hinojosa, J. C., Dapporto, L., Brockmann, E., Dincă, V., Tikhonov, V., Grishin, N., Lukhtanov, V. A., and Vila, R. (2021). Overlooked cryptic diversity in Muschampia (Lepidoptera: Hesperiidae) adds two species to the European butterfly fauna. *Zoological Journal of the Linnean Society*, 193(3):847–859.

Ilyina, E. V. and Morgun, D. V. (2010). Ecological and faunistic review of butterflies (Lepidoptera, Hesperioidea et Papilionoidea) of Daghestan: Part 1. *Entomological Review*, 90(9):1167–1191.

Ilyina, E. V. and Morgun, D. V. (2011). Ecological and faunistic review of butterflies (Lepidoptera, Hesperioidea et Papilionoidea) of Daghestan: Part 2. *Entomological Review*, 91(4):450–466.

John, E., Bağlar, H., Başbay, O., Konstantinou, G., Salimeh, M., and Wiemers, M. (2022). Confirmation of the presence of nominotypical Papilio demoleus demoleus Linnaeus, 1758 (Lepidoptera: Papilionidae) in Cyprus, with additional notes on breeding and potential colonization. *Entomologist's Gazette*, 73(2):117–128.

John, E. and Makris, C. (2022). *Butterflies of Cyprus : a field guide and distribution atlas.* CABI, Wallingford.

Kuznetsov, G. V. (2009). Materials to study of Papilionoidea butterflies (Lepidoptera) from Volgograd region [in russian]. *Caucasian Entomological Bulletin*, 5(2):257–267.

Kuznetsov, G. V. (2011). Some data about biology Melitaea telona Fruhstorfer, 1908 and Melitaea robertsi uvarovi Gorbunov, 1995 (Lepidoptera: Nymphalidae) on Volgograd region [in russian]. *Caucasian Entomological Bulletin*, 7(1):83–84.

Lukhtanov, V. A. and Pazhenkova, E. A. (2021). The taxa of the Hyponephele lycaon - H. lupina species complex (Lepidoptera, Nymphalidae, Satyrinae): deep DNA barcode divergence despite morphological similarity. *Folia Biologica*, 69(1):11–21.

Mamedova, V. R. (2014). Fauna of day Lepidoptera of Low Daghestan [in Russian]. *Vestnik Adygejskogo gosudarstvennogo universiteta*, 137(2):75–81.

Muñoz Sairot, M. G. and Sánchez Mesa, L. (2019). Nueva subespecie de Melitaea ornata (Christoph, 1893), con la descripción de sus estadios preimaginales (Lepidoptera: Nymphalidae). *Arquivos Entomolóxicos*, 21:5–20.

Russell, P. J. C. and Vane-Wright, R. I. (2022). papilio hermione Linnaeus, type species of Hipparchia Fabricius (Lepidoptera, Satyrinae): restoring stability to the application of these names. *Nota Lepidopterologica*, 45:279–294.

Sánchez Mesa, L. and Muñoz Sairot, M. G. (2017). Melitaea ornata (Cristoph, 1893), nueva especie para la Península Ibérica. primeros datos de su morfología, biología y ecología comparada con los de Melitaea phoebe (Denis & Schiffermüller, 1775). (Lepidoptera: Nymphalidae). *Arquivos Entomolóxicos*, 18:313–324.

Wiemers, M., Balletto, E., Dincă, V., Faltynek Fric, Z., Lamas, G., Lukhtanov, V., Munguira, M. L., van Swaay, C. A. M., Vila, R., Vliegenthart, A., Wahlberg, N., and Verovnik, R. (2018). An updated checklist of the European butterflies (Lepidoptera, Papilionoidea). *ZooKeys*, 811:9–45.

Wiemers, M., Chazot, N., Wheat, C., Schweiger, O., and Wahlberg, N. (2020). A complete time-calibrated multi-gene phylogeny of the European butterflies. *ZooKeys*, 938:97–124.

Zhang, J., Brockmann, E., Cong, Q., Shen, J., and Grishin, N. V. (2020). A genomic perspective on the taxonomy of the subtribe Carcharodina (Lepidoptera: Hesperiidae: Carcharodini). *Zootaxa*, 4748(1):182–194.
